# Recent advances in metallic transition metal dichalcogenides as electrocatalysts for hydrogen evolution reaction

**DOI:** 10.1016/j.isci.2022.105098

**Published:** 2022-09-08

**Authors:** Yeoseon Sim, Yujin Chae, Soon-Yong Kwon

**Affiliations:** 1 Department of Materials Science and Engineering & Center for Future Semiconductor Technology (FUST), Ulsan National Institute of Science and Technology (UNIST), Ulsan 44919, Korea

**Keywords:** Catalysis, Materials science, Nanomaterials

## Abstract

Layered metallic transition metal dichalcogenides (MTMDs) exhibit distinctive electrical and catalytic properties to drive basal plane activity, and, therefore, they have emerged as promising alternative electrocatalysts for sustainable hydrogen evolution reactions (HERs). A key challenge for realizing MTMDs-based electrocatalysts is the controllable and scalable synthesis of high-quality MTMDs and the development of engineering strategies that allow tuning their electronic structures. However, the lack of a method for the direct synthesis of MTMDs retaining the structural stability limits optimizing the structural design for the next generation of robust electrocatalysts. In this review, we highlight recent advances in the synthesis of MTMDs comprising groups VB and VIB and various routes for structural engineering to enhance the HER catalytic performance. Furthermore, we provide insight into the potential future directions and the development of MTMDs with high durability as electrocatalysts to generate green hydrogen through water-splitting technology.

## Graphical abstract

**Figure:**
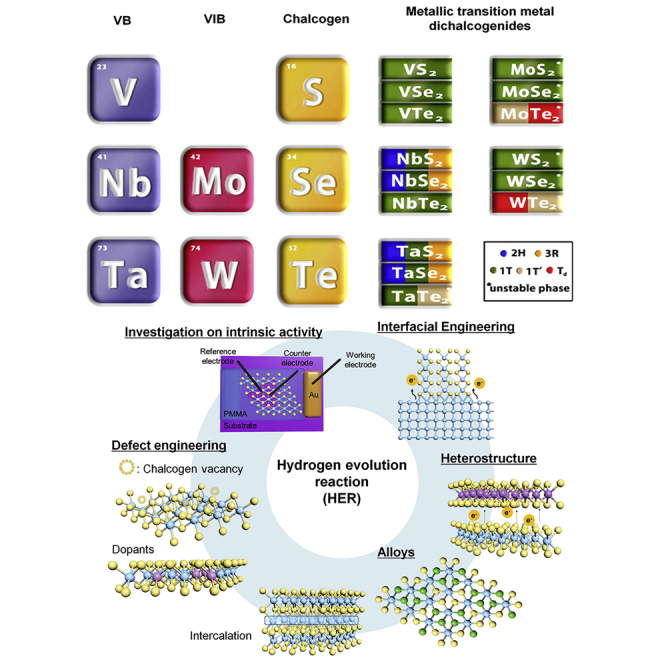

## Introduction

The development of renewable energy technologies has become indispensable given the increasing energy issues related to energy security, environmental pollution, and sustainable economy (Dresselhaus and Thomas, 2001). Hydrogen, which has diverse advantages such as high energy density (142MJkg^−1^), safety, and recyclability, has emerged as a promising energy carrier for achieving zero carbon emissions. The electrochemical reaction from water has also gained attention as a sustainable method for generating green hydrogen; however, the ultimate potential in hydrogen evolution reaction (HER) is yet to be accomplished owing to the use of scarce and expensive precious metals as electrocatalysts (Turner, 1999). Several attempts have been made to solve this problem by lowering the Pt content of the electrocatalysts while maintaining high HER activity (Cheng et al., 2016; Lin et al., 2017). Because of the low utilization efficiency, not all Pt atoms in a typical Pt-based catalyst are active, and Pt single atom-based catalysts tend to agglomerate during catalytic processes, leading to a decrease in the HER activity (Gao et al., 2017). Thus, there is a need for designing novel low-cost, earth-abundant electrocatalyst based on non-precious metals possessing high HER activity and long-term stability.

Various transition metal-based materials, including chalcogenides, phosphides, nitrides, carbides, and oxides, have been extensively researched and predicted to be high-performing HER catalysts (Bhat et al., 2021; Jaramillo et al., 2007; Popczun et al., 2013; Wazir et al., 2022). In particular, layered transition metal dichalcogenides (TMDs) have been recognized as excellent substitutes for Pt-based groups owing to their outstanding chemical stability and theory-guided discovery, which indicate their high HER activity (Hinnemann et al., 2005). Hinnemann et al. revealed that the Mo(10–10) edge in MoS_2_ have a Gibbs-free energy of hydrogen adsorption (Δ*G*_H∗_) of 0.08 eV, which indicates near optimal binding energies of reaction (Hinnemann et al., 2005). Jaramillo et al. first demonstrated by electrocatalytic measurements that the catalytic efficiency of 2H-MoS_2_ exhibits a strong linear dependence on the number of Mo edge sites (Jaramillo et al., 2007). Owing to the inactive MoS_2_ basal plane with a Δ*G*_H∗_ of 1.82 eV, the researchers have studied one strategy for exposing several edges on the restricted area (Kibsgaard et al., 2012; Ye et al., 2016). However, they faced limitations such as low conductivity and physical issues related to catalyst overloads that cause a decrease in charge and mass transport (Benck et al., 2014).

**Figure 1:**
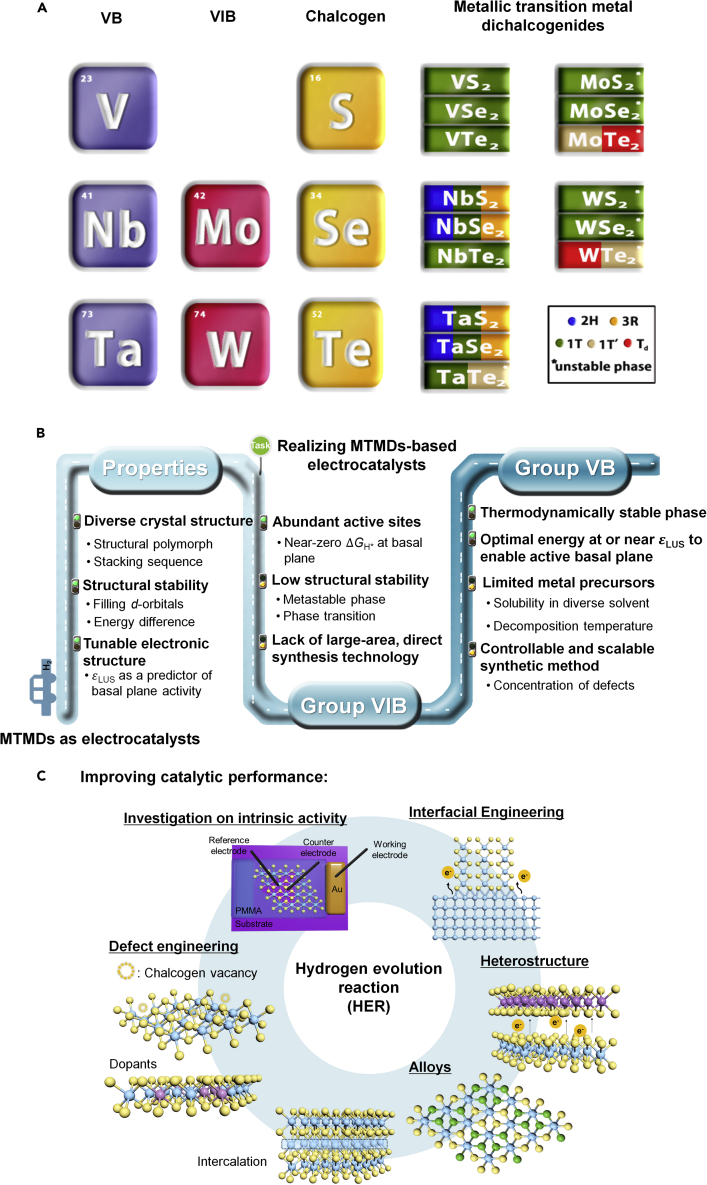
[Overview of MTMDs] (A) A variety of MTMD compounds comprising group VB and VIB. The phase with a low energy state when multiple polymorphs exist is listed from the left. The asterisk (∗) indicates the thermodynamically unstable phase. (B) Challenges and opportunities in MTMDs as electrocatalysts. (C) Strategies to improve catalytic performance of MTMDs-based electrocatalysts.

Of interest, the focus has shifted to another attractive strategy: increasing the intrinsic activity by tuning the electronic structures (Li et al., 2016b; Tang and Jiang, 2016). Some studies demonstrated that MTMDs with intriguing electrical properties such as 1T-MoS_2_ (Voiry et al., 2013a) and 2H-TaS_2_ (Shi et al., 2017) can be Pt-like HER electrocatalysts and hold better HER performance than semiconducting TMDs (STMDs, such as MoS_2_, WS_2_ and MoSe_2_ having H phase). The progress of group VB and VIB MTMDs as shown in Figure 1 indicates the potential to surmount the above-mentioned issues, and it has opened a new route for engineering future electrocatalysts. From the synthesis point of view, the “bottom-up” approaches for scalable production can trigger the feasibility to be applied as MTMDs-based catalytic materials in a practical water-splitting system. However, recent studies on MTMDs are lacking, compared to those on STMDs that are easily accessible by bottom-up synthesis including chemical vapor deposition (CVD) and solution-based reaction. Furthermore, phase transition and post-treatment of as-synthesized MTMDs continue to be explored for realizing improved catalytic performance because the controllable one-step synthesis of MTMDs is limited in relation to structural stability and the adoption of a precursor.

We briefly introduce fundamental and structural features of TMDs that have various polymorphs; however, in this review, we focus on MTMDs in group VB and group VIB. The review aims to (1) highlight extraordinary properties of MTMDs for use as electrocatalysts, (2) condense strategies for the growth of MTMDs, (3) summarize the key technologies of engineering to enhance catalytic performance, and (4) present challenges that remain for MTMDs to become a rising star in the developing efficient electrocatalysts to produce green hydrogen.

### Crystal structure and electronic structure of TMDs

TMDs have a chemical formula of MX_2_ with a transition metal (M = group IVB to VIIIB) layer sandwiched between the two layers of chalcogen (X = S, Se, and Te) atoms. Transition metal (M)-chalcogen (X) atoms combine strong covalent bonds within the layer, and each layer is weakly coupled by the van der Waals force. The fascinating properties of TMDs are strongly related to their crystal and electronic structures (Liu et al., 2017; Mak et al., 2010; Splendiani et al., 2010; Wilson et al., 1974).

### Crystal structure of TMDs

**Figure 2:**
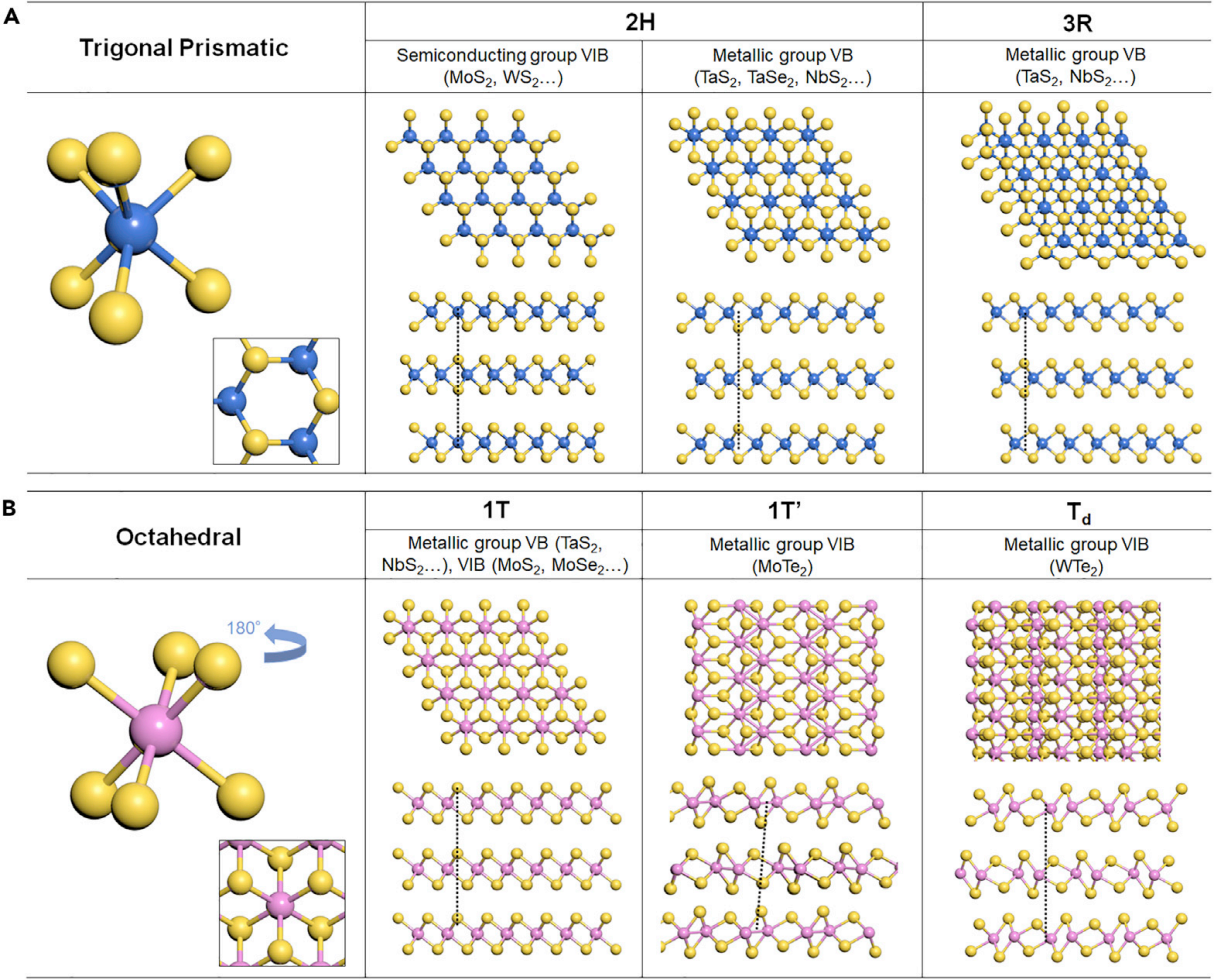
[Crystal structure of TMDs] (A) H- and (B) T- phases.

TMDs show a variety of structural polymorphs. The structure of TMDs depends on the ionicity of the bonding between the transition metal and chalcogen atoms, and the stacking sequence of two or more individual layers with the same symmetry (Gamble, 1974; Huisman et al., 1971; Madhukar, 1975). Figure 2 shows two primary structures of TMDs with the ball-and-stick atomic models: trigonal prismatic (H phase) and octahedral (T phase) coordination. In the H phase for the hexagonal symmetry (Figure 2A), the six X atoms are arranged symmetrically in the upper and lower tetrahedrons with the M atoms as the symmetrical point. The 2H and 3R phases are identified based on sequence stacking of the 1H layer. The two layers with AB stacking as a unit cell in the 2H phase show a hexagonal symmetry (point group D_3h_), whereas the 3R phase composed of three layers with ABC stacking exhibits a rhombohedral symmetry (point group C_3v_). Another major structure, the T phase shown in Figure 2B, is formed by rotating the upper (or lower) tetrahedron by 180°, which characterizes tetragonal symmetry (point group D_3d_). Furthermore, the dimerization of transition metal atoms can lead to the distortion of the 1T phase including the monoclinic (1T′) and orthorhombic (T_d_) structures.

### Electronic structure and structural stability of TMDs

**Figure 3:**
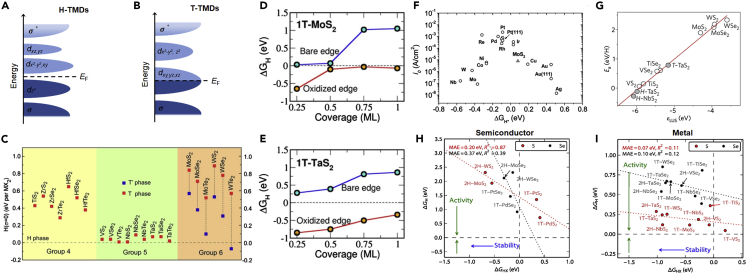
[Effect of electronic structure on structural stability and HER] Schematic for *d*-orbital filling of (A) H and (B) T phases. (C) Energy differences among phases of the monolayer TMDs without mechanical stress. Reprinted with permission from (Hernandez Ruiz et al., 2022). Copyright 2021 The Authors. Small Science published by Wiley-VCH GmbH. Δ*G*_H∗_ as a function of hydrogen coverage for the oxidized (D) 1T-MoS_2_ and (E) 1T-TaS_2_. Reproduced with permission from (Martincová et al., 2020). Copyright 2020 IOP Publishing Ltd. (F) Volcano plot of the exchange current density (*i*_0_) as a function of the Δ*G*_H∗_ for pure metals and MoS_2_. Reproduced with permission from (Jaramillo et al., 2007). Copyright 2007, The American Association for the Advancement of Science. (G) Correlation between the ε_LUS_ descriptor and surface adsorption energy (*E*_a_). Reproduced with permission from (Liu et al., 2017). Copyright 2017, Nature Publishing Group. Plot for the Δ*G*_H∗_ of the basal plane in (H) semiconducting and (I) metallic monolayer TMDs vs. Δ*G*_HX_. Reproduced with permission from (Tsai et al., 2015). Copyright 2015 Elsevier B.V.

The structural stability and electronic structure of TMDs is strongly influenced by the *d* electron counts of the transition metal (Chhowalla et al., 2013; Mattheiss, 1973). The *d*-orbitals of the transition metal in H-TMDs are divided into three degenerated states *d_z___2__*_,_ *d_x___2___-y___2__*_,*xy*_, and *d*_*xz*,*yz*_ with a significant energy gap of ∼1 eV between the *d_z___2__* and *d_x___2___-y___2__*_,_*_xy_* orbitals (Figure 3A). In contrast, the T-TMDs result in *d_z_*__2___,_ *_x_*__2__*_-y_*__2__ (*e*_*g*_), and *d*_*xy*,*yz*,*xz*_ (*t*_*2g*_) crystal field splitting (Figure 3B). The *d*-orbitals are filled from 0 (*d*^*0*^, group IVB) to 6 (*d*^*6*^, group VIIIB) electrons because the oxidation state of the transition metal in the TMD is equal to +4. The *t*_*2g*_ levels of the T-TMDs are situated between the *d_z___2__* and *d_x___2___-y___2__*_,_*_xy_* levels of the H-TMDs. All occupied *d*-orbitals lead to TMDs with a semiconducting nature, whereas the partially occupied *d*-orbitals cause the metallic behavior of TMDs. Under ambient conditions, two electrons in group VIB TMDs tend to fill the *d_z___2__* level (2H) with priority over *t*_*2g*_ levels (1T) because the required energy is lower. They prefer the thermodynamically stable semiconducting-2H phase to the metastable metallic-1T phase, except for WTe_2_, which exists as the T_d_ phase. As shown in Figure 3C, the 1T′ phase possesses even lower ground-state energy without mechanical stress compared to that of the 1T phase (Duerloo et al., 2014; Hernandez Ruiz et al., 2022; Zhang et al., 2016). Therefore, structural distortions by mechanical stress or electron injection have attracted extensive attention for improving the structural stability of group VIB MTMDs. In the case of group VB TMDs, both the 2H phase and 1T are stable because the energy difference between the 2H and 1T phases is less than 0.1 eV. Their *d*-orbitals are partially filled by the additional electron regardless of the crystal field, and therefore, both the 2H and 1T-group VB TMDs are always metallic. Recent studies on group VB MTMDs revealed extended four structures including 2H (Jeong et al., 2012), 3R (Deng et al., 2020), 1T (Fu et al., 2016), and 1T’ (Li et al., 2018a).

After a long time, the oxidation rate of TMDs can be accelerated by reaction with water and oxygen, starting from the edge or the defects, regardless of the phase and chalcogen (Voiry et al., 2013a). Of interest, the spontaneous initial oxidation of MTMDs (e.g., 1T-MoS_2_ and 1T-TaS_2_) can passivate the edge and protect the material against more severe oxidative degradation (Martincová et al., 2020). The adsorbed oxygen atoms eventually form a SO_2_ group binding to one of the edge S atoms, resulting in a thermodynamically favored state with an intermediate S-O-MO structure (Pető et al., 2018). This oxidation is not harmful to HER catalysis in 1T-MoS_2_ but may adversely affect other intriguing functionalities and phenomena reported for MTMDs (Figures 3D and 3E). The structural stability of TMDs is well maintained in acidic and alkaline solutions, both of which are HER-driven environments. Wang et al. demonstrated that the dissolution ratio of MoS_2_ in acid is three orders of magnitude higher than that of TMP (e.g., CoP and MoP) but comparable to that of Pt (Wang et al., 2021). Although the HER performance of MTMDs in alkaline solution has not yet been well explored, they are reported to have poorer activity in the alkaline medium than in an acidic medium. Moreover, several studies have reported similar results regarding the stability in acidic media as well (Park et al., 2020).

### Correlation between electronic structure and hydrogen production

In general, the HER is a multi-step reaction that includes adsorption, reduction, and desorption steps, which are highly dependent on the intrinsic chemical and electronic properties of the electrode surface as well as the electrolyte (Table 1). Most studies have reported that the performance of electrocatalysts in alkaline solutions is inferior to that in acidic solutions (Li et al., 2017a). This is because in alkaline solutions, additional energy is required in the Volmer step to dissociate the water molecules, whereas in the acidic medium, the electrolyte releases protons from the hydronium cation (H_3_O^+^). Three associated descriptors were used to evaluate the ease with which a catalyst initiates the reaction: water adsorption energy (E_ad_), activation energy of water dissociation (E_ac_), and Δ*G*_H∗_. Among the three parameters, Δ*G*_H∗_ is the most commonly known, and it indicates the binding strength of H∗ on the catalyst surface in both acidic and alkaline solutions. According to the Sabatier principle, optimal electrocatalysts for HER can have moderate binding energies of hydrogen adsorption. The Δ*G*_H∗_ of edge in 2H-MoS_2_ —one of the TMDs-based electrocatalysts—is located below the precious metals, which indicates an enormous potential as an electrocatalyst with high activity, as shown in the volcano plot (Figure 3F) (Jaramillo et al., 2007). Recently, it was demonstrated that the basal plane of 1T-MoS_2_ (Voiry et al., 2013a, 2016) and S vacancies with the localized metallic states in 2H-MoS_2_ (Hinnemann et al., 2005) also allow for increasing HER activity. Figure 3G reveals that the energy at or near the lowest unoccupied states (ε_LUS_) has a linear relationship with the corresponding surface adsorption energy (*E*_a_) (Liu et al., 2017). The polymorphism of TMDs is a key origin to achieve a high density of electronic states at the Fermi level, and this promotes the electrode kinetics for HER (Liu et al., 2017; Wang et al., 2017b). Considering the HER mechanism, various approaches have been made to develop outstanding electrocatalysts that have high intrinsic activity and a large active surface area, in addition to allowing fast charge transfer and exhibiting prolonged electrochemical stability. Among them, group VB TMDs possess *ε*_LUS_ (≈ –6 eV) that correspond to near-zero *E*_a_ values. Moreover, the metallic conductivity of their stable polymorphs and the presence of active sites in their basal plane enable improved catalytic activity and faster reaction kinetics. Therefore, they show higher potential as efficient and durable electrocatalysts than do group VIB STMDs. Similarly, Figures 3H and 3I indicate the plot of the Δ*G*_H∗_ as a function of the Gibbs-free energy of HX adsorption (Δ*G*_HX∗_) at the basal planes of the semiconducting and metallic TMDs (Tsai et al., 2015); thus, a more stable electrocatalyst requires higher H-X binding. This is because the X atoms in TMDs can withdraw electrons from the transition metals owing to their higher electronegativity, and X can act as the active site to stabilize the reaction intermediates. Figures 3G–3I theoretically demonstrate that the metallic basal plane of the TMD plays a more critical role in modulating the HER activity than do the structure and composition. In this review, we focus on MTMDs-based electrocatalysts.

**Table 1:** General HER mechanism in acid and alkaline solution

	Acid	*A*lkaline
**Overall**	∗ + 2H^+^ + 2e^−^ → H_2_	∗ + 2H_2_O + 2e^−^ → H_2_ + 2OH^−^
**Volmer**	∗ + H^+^ + e^−^ → H∗	∗ + H_2_O + e^−^ → H∗ + OH^−^
**Herovsky**	∗ + H^+^ + e^−^ + H∗ → H_2_ +∗	∗ + H_2_O + e− + H∗ → H_2_ + OH- +∗
**Tafel**	2H∗ → H_2_ + 2∗	2H∗ → H_2_ + 2∗

∗ denotes an active site on the surface of an electrocatalyst.

### Synthesis of MTMDs

Undoubtedly, the controllable synthesis of MTMDs with a high crystalline quality, thickness uniformity, large domain size, and continuity is critical not only to manipulate electronic structure but also extensively investigate catalytic properties and unique physical properties such as charge density wave (CDW) (Xi et al., 2015), superconductivity (Navarro-Moratalla et al., 2016), and ferromagnetism (Zhao et al., 2020). The controllable synthesis of group VB MTMDs and metastable group VIB MTMDs remains quite challenging although several well-established techniques have been developed for producing high-quality group VIB TMDs with H (Kim et al., 2019), T′, and T_d_ (Song et al., 2020) phase. In this section, we will provide an overview of the synthesis strategies to achieve high-quality MTMDs-based electrocatalysts.

### Top-down approach

**Figure 4:**
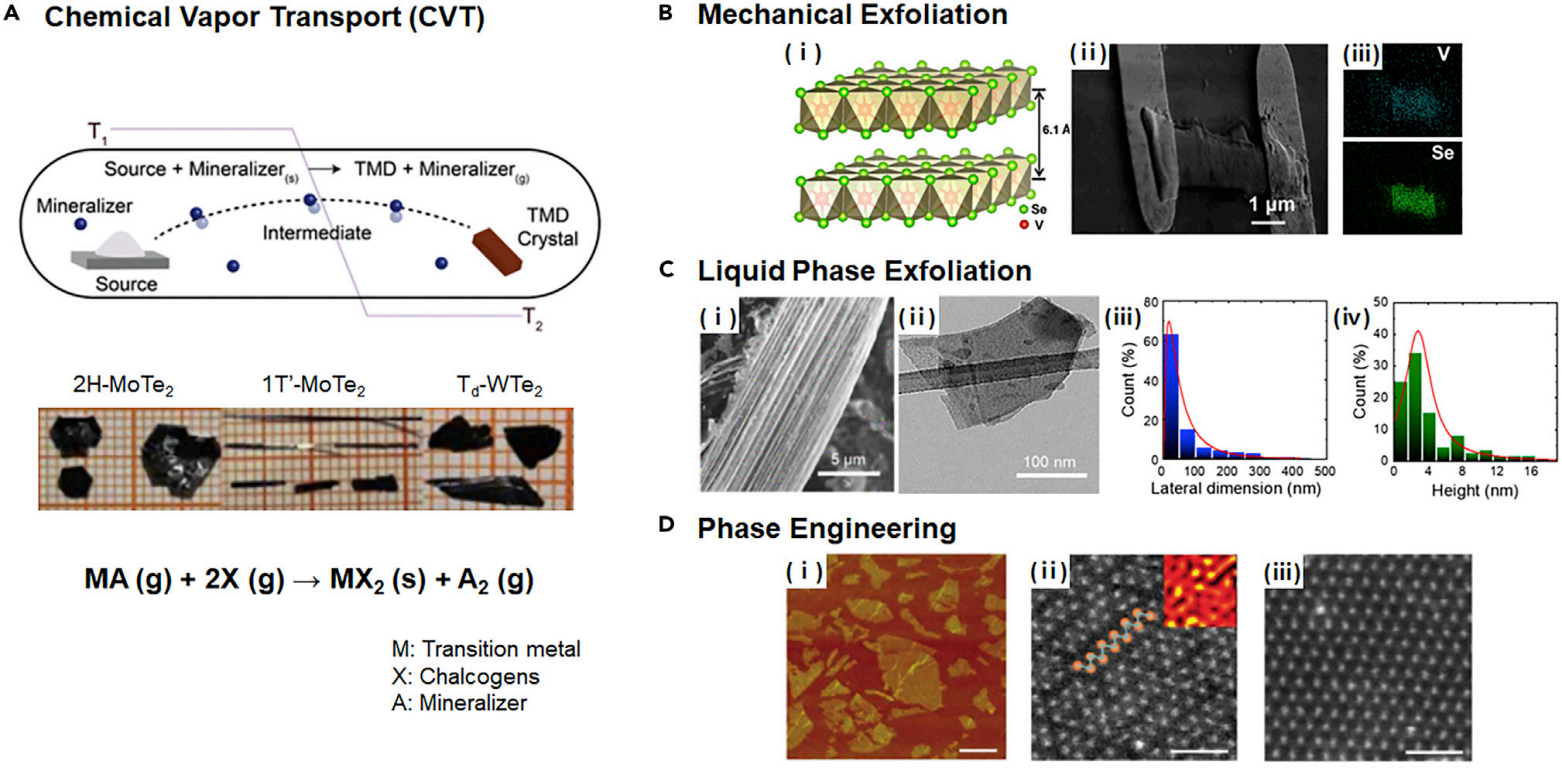
[Top-down approach for the preparation of MTMDs] (A) Schematic of the single crystal growth of bulk TMDs by CVT, and photographs of the single crystals of 2H-MoTe_2_, 1T′-MoTe_2_, and T_d_-WTe_2_. Upper image in (A) Reprinted with permission from (Wang et al., 2019). Copyright 2019 WILEY-VCH Verlag GmbH & Co. KGaA, Weinheim. Lower image in (A) reproduced with permission from (Lv et al., 2017). Copyright 2017, The Authors, published by Springer Nature. (B–D) Typical three isolation methods of low-dimensional MTMDs from bulk TMDs grown by CVT. (B) 1T-VSe_2_ nanosheet held by a scotch-tape based mechanical exfoliation method. (i) Schematic of the atomic model of VSe_2_ (V: red, Se: bright green). (ii) SEM image and (iii) corresponding elemental mapping of a representative VSe_2_ nanosheet with Au electrode. Reprinted with permission from (Yan et al., 2017). Copyright 2017, American Chemical Society. (C) Exfoliated 2H-TaS_2_ flake obtained using ultrasonication and dispersion. (i) High magnification SEM image of edges in bulk 2H-TaS_2_ grown by CVT, which indicates a layered structure. (ii) TEM image of 2H-TaS_2_ flake chemically exfoliated from bulk 2H-TaS_2_ in (i). Statistical analysis of (iii) the lateral dimension and (iv) height of 2H-TaS_2_ flakes. Reprinted with permission from (Najafi et al., 2020). Copyright 2020, American Chemical Society. (D) 2H to 1T phase transformation of the WS_2_ flakes via Li-intercalation. (i) Representative AFM image of exfoliated WS_2_. HAADF-STEM images of a chemically exfoliated WS_2_ monolayer showing regions of (ii) 1T and (iii) 2H structures. Reprinted with permission from (Voiry et al., 2013b). Copyright 2013, Nature Publishing Group.

The top-down method is a leading route to obtain low-dimensional, supreme quality TMDs single crystal. Most investigated novel physical properties of TMDs are demonstrated in exfoliated flakes from bulk crystals (Geim and Grigorieva, 2013). Chemical vapor transport (CVT) is typical approach for the single crystal growth of TMDs in the bulk foam (Figure 4A) (Wang et al., 2019). MX_2_ structures can be produced in various compounds by reacting transition metals and chalcogens with a selected mineralizer, as shown in the inset of Figure 4A (Lv et al., 2017). Thermodynamically stable group VB MTMDs can be easily obtained by mechanical/chemical exfoliation from bulk group VB MTMDs. Yan et al. produced metallic multilayered VSe_2_ nanosheets by Scotch-tape-based mechanical exfoliation and the evaluated tunability of HER performance by applying a back gate voltage (Figure 4B) (Yan et al., 2017). Owing to the limited sample size and poor production rate, this approach is incompatible with a large-area synthesis of MTMDs. Instead, liquid phase exfoliation (LPE) has been advanced for the scalable production of MTMD films (Lin et al., 2018). In this process, the interlayer spacing of MTMDs is expanded by the insertion of external ions such as Li-ion and ammonium ion. In addition, the expanded MTMDs are laminated using an external mechanical driving force. As shown in Figure 4C, Najafi et al. obtained few-layer H-TaS_2_ and H-TaSe_2_ flakes with a lateral size of 10–450 nm by LPE via 2-propanol (Najafi et al., 2020). The high-quality H-TaS_2_ flakes formed by batch production act as outstanding electrocatalysts for HER.

The synthesis of metastable group VIB TMDs in the bulk requires higher formation energy as compared to the stable 2H-group VIB TMDs. Phase transition via external force (charge transfer (Kang et al., 2014), electric field (Shang et al., 2019), and mechanical stress (Duerloo et al., 2014)) is a major approach to stabilize a metastable phase. In 2013, Voiry et al. reported that exfoliated monolayer WS_2_ with a high concentration of metallic 1T-edges using a Li-intercalated LPE method served as an efficient electrocatalyst for hydrogen evolution (Figure 4D) (Voiry et al., 2013b). In the LPE process, the as-prepared 2H-WS_2_ powder was Li intercalated to form Li_*x*_WS_2_. Although there is a sufficiently large energy barrier in the 2H to 1T phase transition, their energy barrier is unsubtly lowered with the assistance of Li^+^ intercalation (Xia et al., 2017); it is attributed to modulating the electron injection from a semiconducting to a metallic one via Li-intercalation. Therefore, they obtained the as-exfoliated WS_2_ nanosheets with zigzag-like local distorted lattice configuration by strain as shown in the high-angle annular dark-field scanning transmission electron microscope (HAADF-STEM) image (the middle of Figure 4D).

### Bottom-up synthesis

The control of the process and crystal quality of obtained samples are poor although the LPE method provides feasibility for the batch production of MTMDs. Many researchers are investigating strategies to develop a scalable production of high-quality MTMDs. Bottom-up synthesis is the most notable way to increase their potential application and practical utilization. Recent studies revealed that some metastable group VIB TMDs may be directly synthesized via solution-based reaction and chemical vaporization. However, the reliable method used to attain group VIB MTMDs is in the early stages of the study. Furthermore, the synthesis of group VB MTMDs is limited because the number of available M precursors in group VB is small and most of them have high melting points (Table 2). In this section, we aim to provide the current status of the bottom-up synthesis of MTMDs.

**Table 2:** Melting temperatures and solubility of TMD precursors

	Element	Precursor	Melting point (°C)	Solubility in water (g/L)
Group VB	V	NH_4_VO_3_	200	4.8 g/mL ∗Soluble in diethanolamine, ethanolamine
		NaVO_3_	630	19.3 g/100mL (at 20°C)
		Na_3_VO_4_·10H_2_O	858	22.17 g/100 mL
		VO(acac)_2_ (C_10_H_14_O_5_V)	258	Negligible ∗Soluble in ethanol and benzene
		VCl_3_	>300	Decompose
		V_2_O_5_	690	8.0 g/L
	Nb	NbCl_5_	204	Decompose
		Nb_2_O_5_	1,512	Insoluble ∗Soluble in HF
	Ta	TaCl_5_	216	Decompose ∗Soluble in absolute alcohol and KOH
		Ta_2_O_5_	1,872	Negligible ∗Insoluble in organic solvents and most mineral acid, reacts with HF
Group VIB	Mo	(NH_4_)_2_MoS_4_	155	Highly soluble ∗Sparingly soluble in ethanol
		(NH_4_)_6_Mo_7_O_24_·4H_2_O	90	65.3 g/100 mL
		Na_2_MoO_4_·2H_2_O	687	84 g/100 mL (at 100°C)
		MoCl_5_	194	Hydrolyzes ∗Soluble in organic solvents
		MoO_3_	795	4.9 g/L
	W	(NH_4_)_2_WS_4_	–	–
		(NH_4_)_6_H_2_W_12_O_40_·4H_2_O	100	–
		Na_2_WO_4_	698	74.2 g/100mL ∗Slightly soluble in ammonia, insoluble in alcohol, acid
		WCl_6_	275	Hydrolyzes ∗Soluble in chlorocarbons
		WO_3_	1,473	Insoluble ∗Slightly soluble in HF
Chalcogen	S	C_2_H_5_NS (thioacetamide)	115	16.3
		CH_4_N_2_S (thiourea)	182	137 g/L
		C_3_H_7_NO_2_S (l-cysteine)	240	Soluble ∗Soluble in ethanol
	Se	Se powder	221	Insoluble
		SeO_2_	340	39.5 g/100 mL ∗Soluble in ethanol, acetone, acetic acid
		(PhCH_2_)_2_Se_2_ (C_14_H_14_Se_2_)	91	4.32 g/L
	Te	Te powder	449	Insoluble ∗Soluble in acid, potassium hydroxide

### Solution-based method

**Figure 5:**
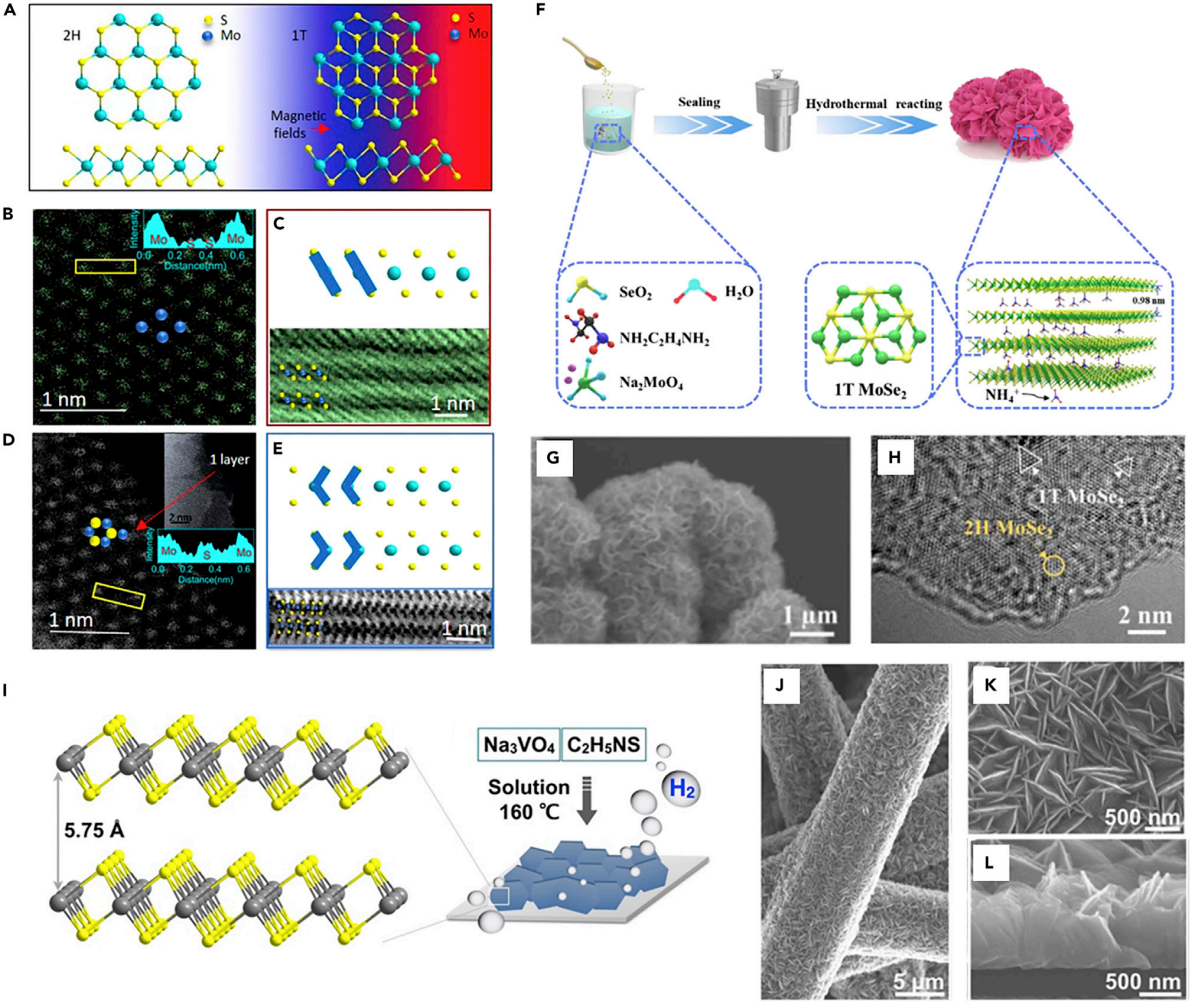
[Solution-based preparation of MTMDs] (A–E) 1T-rich MoS_2_ crystal grown by magnetic field. (A) Schematic of the atomic model of MoS_2_. (B) The plan-view HAADF-STEM and (C) cross-sectional ABF images of the single-layer of the 9T magneto-hydrothermally grown MoS_2_ (MoS_2_-9T), which show the 1T phase lattice configuration. (D) The plan-view HAADF-STEM and (E) cross-sectional ABF images of MoS_2_-0T showing the 2H phase. Reprinted with permission from (Ding et al., 2019). Copyright 2019, American Chemical Society. (F–H) Synthesized MoSe_2_ with a rich 1T phase via NH_4_^+^ intercalation. (F) Schematic for the preparation of NH_4_^+^-intercalated 1T-rich MoSe_2_. Typical (G) SEM and (H) HRTEM images of expanded 1T-rich MoSe_2_. Reprinted with permission from (Zhou et al., 2021). Copyright 2021, American Chemical Society. (I–L) Vertical 1T-VS_2_ nanoplate grown via hydrothermal processing. (I) Atomic structure and schematic exhibiting the growth procedure of 1T-VS_2_ nanoplates. (J) Low-magnification, (K) high-magnification top-view, and (L) side-view SEM images of 1T-VS_2_ nanoplates. Reprinted with permission from (Liang et al., 2016). Copyright 2016, American Chemical Society.

Post-treated group VIB MTMDs using a conventional method such as liquid exfoliation (Voiry et al., 2013b), electron-beam irradiation (Cho et al., 2015), mechanical strains (Duerloo et al., 2014), and plasmonic hot electrons (Kang et al., 2014) are transformed readily into the stable 2H-phase via intermediates and oxidation. A recent study reported that ambient stable 1T-MoS_2_ and 1T-WS_2_ for more than 1 year were synthesized using the facile hydrothermal method under a magnetic field that optimizes the kinetics reaction (Figure 5A) (Ding et al., 2019). The hydrothermal method refers to a heterogeneous reaction that depends on solubility in water or an organic solvent in a sealed steel container with Teflon liners. The reaction occurs in a low-temperature range of 100–200°C under the pressure generated by the container. However, Ding et al. applied a magnetic field that can transfer high energy on an atomic scale of the substance in addition to a general hydrothermal method. The HAADF-STEM images of the hydrothermally synthesized MoS_2_ at 9 T and 0 T illustrate the difference in the atomic configuration (Figures 5B–5E). Figures 5B and 5C show the 1T-phase as judged from the intensity profile, which indicates that S atoms are dispersed uniformly around the Mo atoms. In contrast, the intensity variations of 2H-phase are detected, wherein the two duplicating sulfur atoms amplify the signal along the electron beam direction (Figures 5D and 5E); these results indicate that the stable 1T-(Mo, W)S_2_ originates from the enhanced kinetics. Further, Zhou et al. presented the formation of 1T-MoSe_2_ nanosheets via interaction with charged reaction by-product (Zhou et al., 2021). Figure 5F depicts the synthetic procedure of the expanded 1T-rich MoSe_2_ nanosheet using ethylenediamine (NH_2_C_2_H_4_NH_2_) that plays a critical role during hydrothermal reaction. NH_2_C_2_H_4_NH_2_ is decomposed NH_4_^+^, and then generated NH_4_^+^ intercalates into MoSe_2_. As shown in Figures 5G and 5H, NH_4_^+^-intercalated 1T-rich MoSe_2_ nanosheets were obtained because the 1T structure is stabilized by charge transfer from NH_4_^+^ to MoSe_2_.

Compared to group VIB MTMDs, the synthesis of thermodynamically stable group VB MTMDs using a solution-based method is relatively easy; however, few studies have been performed except for VX_2_ because of the low solubility of the group VB precursor in water and organic solvents (Table 1). Figure 5I displays the grown 1T-VS_2_ nanoplates by hydrothermally reacting Na_3_VO_4_·10H_2_O that has a high solubility in water (221.7gL^−1^ at room temperature as summarized in Table 1), reported by Liang et al. (Liang et al. (2016)). The representative Scanning electron microscopy (SEM) images in Figures 5J–5L indicate that 1T-VS_2_ nanoplates with ∼30 nm in thickness and ∼800 nm lateral dimension were grown uniformly, and they fully cover the skeletons of the carbon paper. They suggested that the low cost and scalable solution synthesis of VS_2_ catalyst can enable a promising electrocatalysts for large-scale hydrogen production.

### Chemical vapor deposition method

**Figure 6:**
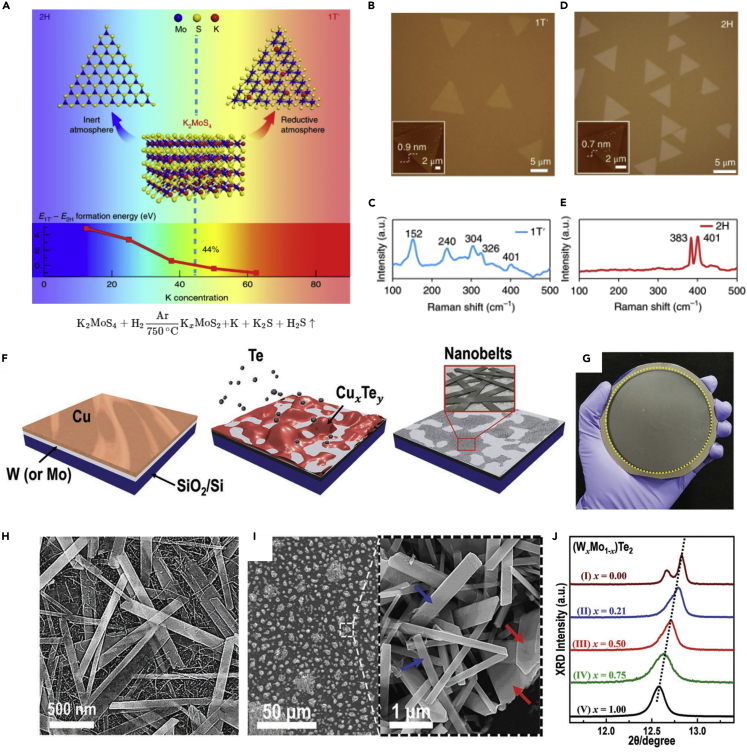
[Growth of group VIB MTMDs using CVD] (A–E) Phase-controlled synthesis of MoS_2_ flakes using potassium. (A) Schematic for phase-controlled synthesis of MoS_2_ and formation energy difference between 2H and 1T′ phase. (B) OM image and (C) Raman spectra of 1T′-MoS_2_ monolayers. (D) OM image and (E) Raman spectra of CVD-grown 2H-MoS_2_ monolayers. Reprinted with permission from (Liu et al., 2018b). Copyright 2018, The Authors, under exclusive licence to Springer Nature Limited. (F–J) Eutectic metal alloy-assisted synthesis of T_d_-WTe_2_ nanobelts. (F) Schematic of the tellurization process. (G) Photograph of grown WTe_2_ nanobelt on a 4 in. SiO_2_/Si wafer. Representative SEM images of (H) WTe_2_ and (I) MoTe_2_ nanobelts. (J) Typical XRD pattern of (W_*x*_Mo_1-*x*_)Te_2_ nanobelts. Reprinted with permission from (Kwak et al., 2018). Copyright 2018 WILEY-VCH Verlag GmbH & Co. KGaA, Weinheim.

CVD has been widely used to perform synthesis of high-quality TMDs. The morphology, thickness, and defect in CVD-grown TMDs can be elaborately controlled because of the broad tunability of their substrates and growth parameter (including precursor, growth temperature, working pressure, growth time and carrier gas) (Kim et al., 2019; Lee et al., 2020). Group VIB MTMDs have achieved only MX_2_ materials with a relatively stable 1T′ structure. In 2018, Liu et al. initially reported the direct synthesis of 1T′-MoS_2_ monolayers with high purity and superior quality using a one-step CVD process (Liu et al., 2018b). Figure 6A exhibits that the effect of intermediate K_*x*_MoS_2_ formed during the process on the phase stability of the MoS_2_ monolayers using the density functional theory (DFT) calculation. As shown in the reaction formula (Figure 6A), the injected H_2_ gas plays a critical role in building a reductive atmosphere. Therefore, 1T′-MoS_2_ triangular thin flakes are synthesized successfully in the mixture of H_2_ and Ar (Figure 6B). Figure 6C shows five Raman peaks demonstrating a 1T′-MoS_2_ nature. In pure Ar, high-quality triangular 2H-MoS_2_ monolayers (Figure 6D) are obtained, as revealed by the Raman spectra (Figure 6E). Kwak et al. reported a novel scalable process to obtain single-crystalline MTe_2_ (M: W, Mo) nanobelts on the desirable substrates at low temperature (≤ 500°C) and short growth time (≤ 10 min) (Kwak et al., 2018). The production of a high-quality stoichiometric MTe_2_ layer with spatial homogeneity is limited because of the low activity of tellurium during process and structural instability by oxidation under ambient environment (Naylor et al., 2016). Eutectic alloy (e.g., Cu_*x*_Te_*y*_) was employed as a Te precursor to synthesize high-quality MTe_2_ nanobelts, as indicated in Figure 6I. Single crystalline T_d_-WTe_2_ nanobelts on a 4 in. SiO_2_/Si were synthesized using this novel approach. The structural and surface analyses (Figures 6K–6M) indicate that the phase, composition, and dimensionality of all MTe_2_ crystals are manipulated using the control process based on the growth parameter (such as temperature and time). Furthermore, the MTe_2_ nanobelts can be directly synthesized onto a targeted surface. Then, Sim et al. demonstrated that eutectic alloy-assisted growth is a promising method for the scalable production of MTMDs-based electrocatalysts, which presents the structural controlled W-based TMDs electrocatalysts with efficient HER activity via this method (Sim et al., 2021).

**Figure 7:**
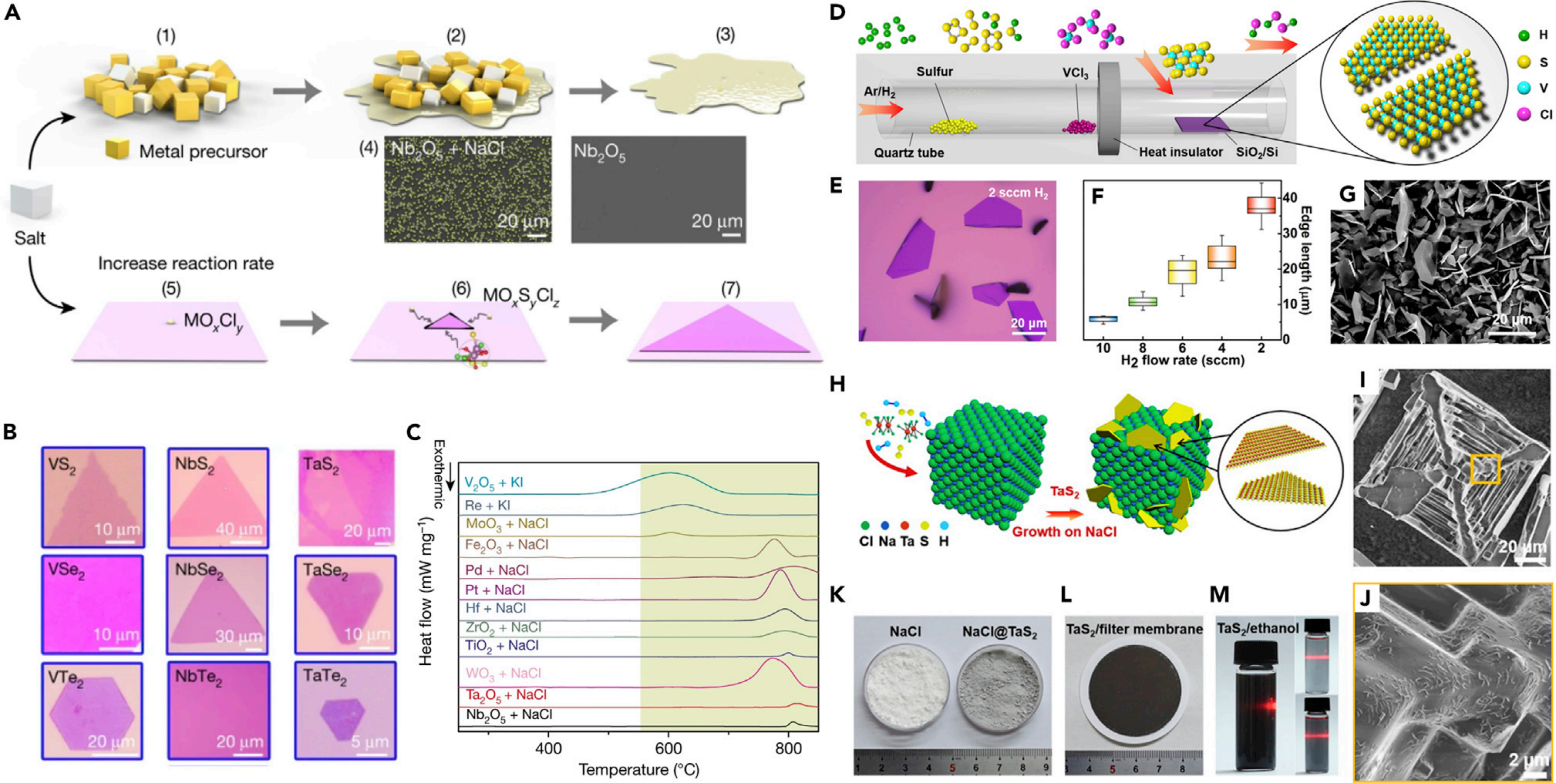
[Growth of group VB MTMDs using CVD] (A–C) Molten-salt-assisted CVD for the growth of TMDs. (A) Schematics of CVD process. (1–3) Decrease in the melting point of the transition metal precursor because of the added salt. (4) Representative SEM images of the Nb nucleus with and without added salt. (5–7) Synthetic procedure of the TMDs layer with the intermediate product. (B) Representative OM images of group VB TMDs synthesized using molten salt. (C) TG-DSC curve of salts mixed with the transition metal precursors. Reprinted with permission from (Zhou et al., 2018a). Copyright 2018, Macmillan Publishers Ltd., part of Springer Nature. (D–G) Synthesized 1T-VS_2_ nanosheets using transition metal chloride. (D) Scheme of CVD setup. (E) OM image of 1T-VS_2_ grown under 100 sccm Ar mixed 2 sccm H_2_. (F) Plot of 1T-VS_2_ edge length versus H_2_ flow rate. (G) Typical SEM image of the high-density VS_2_ nanosheet. Reprinted with permission from (Ji et al., 2017). Copyright 2017, American Chemical Society. (H-M) Scalable production of 1T-TaS_2_ using NaCl templates. (H) Schematic for the growth of 1T-TaS_2_ flakes on the NaCl powder via APCVD. (I) Representative SEM image of 1T-TaS_2_ nanosheets on the NaCl crystal. (J) Magnified SEM image from the yellow square of (I). Photographs of (K) NaCl powder before and after the process, (L) TaS_2_/filter membrane after vacuum filtration, and (M) the dispersed 1T-TaS_2_ nanosheets in ethanol as a function of concentration. Reprinted with permission from (Huan et al., 2019). Copyright 2019, American Chemical Society.

Recently, the CVD method revealed considerable potential in the growth of group VB MTMD with a large domain size and a controllable phase (Deng et al., 2020; Shi et al., 2017). However, oxides of groups VB and VIB showed relatively high melting points, which inhibit the decomposition of M precursors through thermal annealing (Table 1). There are two methods to overcome this issue: (1) An alkali halide-assisted method and (2) the use of transition metal chlorides with lower melting point as an M precursor. In 2018, Zhou et al. discovered that molten salt can widely decrease the melting points of diverse transition metal oxides. The growth mechanism of molten-salted CVD method is illustrated in Figure 7A (Zhou et al., 2018a). As a representative example, the SEM images in Figure 7A show a comparison of the observed Nb nucleus with and without salt, which reveals a strong mass flux of the M precursor improved by the salt. Some metal oxides can combine with salt to generate metal oxychlorides; these decompose at a suitable temperature and enable the formation of thin 2D group VB MTMDs nanoflakes (Figure 7B). The result of thermogravimetry and differential scanning calorimetry (TG-DSC) as shown in Figure 7C suggests that the decomposed temperature of salts mixed with all transition metal oxides reduced within the temperature window from 600°C to 850°C. Further, the mechanism of the molten-salted CVD process was widely adopted in several studies on the growth of TMDs (Li et al., 2018b; Wang et al., 2017a).

Ji et al. reported that VS_2_ nanosheets were successfully grown using the typical CVD setup using transition metal chloride VCl_3_ (Ji et al., 2017). Figure 7D shows the facile CVD process to grow VS_2_ nanosheets under a mixed Ar/H_2_ gas flow with various substrates. A typical optical microscopy (OM) image in Figure 7E shows thin VS_2_ nanosheets with an edge length of ∼40 μm. Of interest, the morphology of VS_2_ nanosheets is controlled using the H_2_ flow rate. The evolution of the average edge length as a function of the H_2_ flow rate is plotted in Figure 7F, which shows the tunability of the dimension. Their application as electrodes of the electronic device and the energy conversion system is proved using high-dense VS_2_ nanosheets (Figure 7G). In a similar method using TaCl_5_, Shi et al. fabricated thickness-tunable 2H-TaS_2_ flakes and large-area films on Au foil; then, they evaluated the feasibility as electrocatalysts for HER (Shi et al., 2017). The obtained TaS_2_-based electrocatalysts showed high HER performance, which proves that the CVD method serves as a strategy for producing an efficient electrocatalyst. Of interest, Huan et al. reported that NaCl powder as a growth template is an effective electrocatalysts to trigger the scalable synthesis of group VB MTMDs in CVD (Huan et al., 2019). The synthesis process and 3D structure of TaS_2_ on NaCl are indicated in Figure 7H. A considerable amount of large TaS_2_ nanosheets are discovered vertically on the corners and curved surfaces of the micron-sized multilevel NaCl crystals, as indicated by the SEM images in Figures 7I and 7J. After the CVD process, the NaCl crystal powder displayed a visible color change from white (before) to gray (after) (Figure 7K). A purified TaS_2_ nanosheet was achieved easily by dissolving the NaCl crystals in deionized water and then filtrating the solution through a filter membrane (Figure 7L). As indicated in Figure 7M, the filtrated TaS_2_ was directly dispersed in target solvents for subsequent characterization. The fabricated TaS_2_ electrocatalysts via this method exhibited outstanding HER activity. Comprehensive research covering the scalable production, green transfer, and energy-related application of high-quality MTMDs was presented by advancing a novel NaCl template-mediated growth approach.

### Engineering of MTMDs-based electrocatalysts for HER

Many researchers have attempted to develop commercially practical MTMD-based electrocatalysts because of the discovery of 1T-MoS_2_ as the electrocatalysts (Lukowski et al., 2013). The catalytic active site of MTMDs is present at both edges and basal planes, and these materials exhibit superior electrical conductivity and optimal Δ*G*_H∗_ close to zero (Pan, 2014). Group VIB MTMDs have been initially devoted to evolving their HER activities via tuning electronic and structural features (Shi et al., 2018; Sokolikova and Mattevi, 2020). Although group VIB MTMDs have been tremendously explored toward distinguished electrochemical catalysts, critical restrictions such as thermodynamic instability and shortage of controllable synthetic method remain (Yu et al., 2018). Furthermore, structural engineering techniques using group VB MTMDs with structural durability, which include edge engineering (Guo et al., 2020), defect engineering (Zhang et al., 2017b), and interfacial engineering (Gnanasekar et al., 2019), have recently been introduced as another key strategy to accelerate hydrogen production. Under this section, we closely describe the fundamental catalytic properties of MTMDs consisting of group VB and group VIB and focus on comprehending the influence of structural modification on the HER catalytic activities in the MTMDs.

### Intrinsic catalytic

The enhancement of electrocatalytic performance depends on the specific active site of materials that lead to hydrogen adsorption and desorption. Understanding the fundamental differences between natural active sites is critical for studying the mechanism and subsequent designing of catalysts that can accelerate HER activity (Huang et al., 2020; Jiao et al., 2015). Increasing the intrinsic catalytic activity directly results in improved electrode performance in a manner that mitigates the transport issues arising from the higher catalyst loading (Benck et al., 2014). Thus, we investigated the intrinsic activity of the catalyst on a per-site basis.

### Group VIB MTMDs

**Figure 8:**
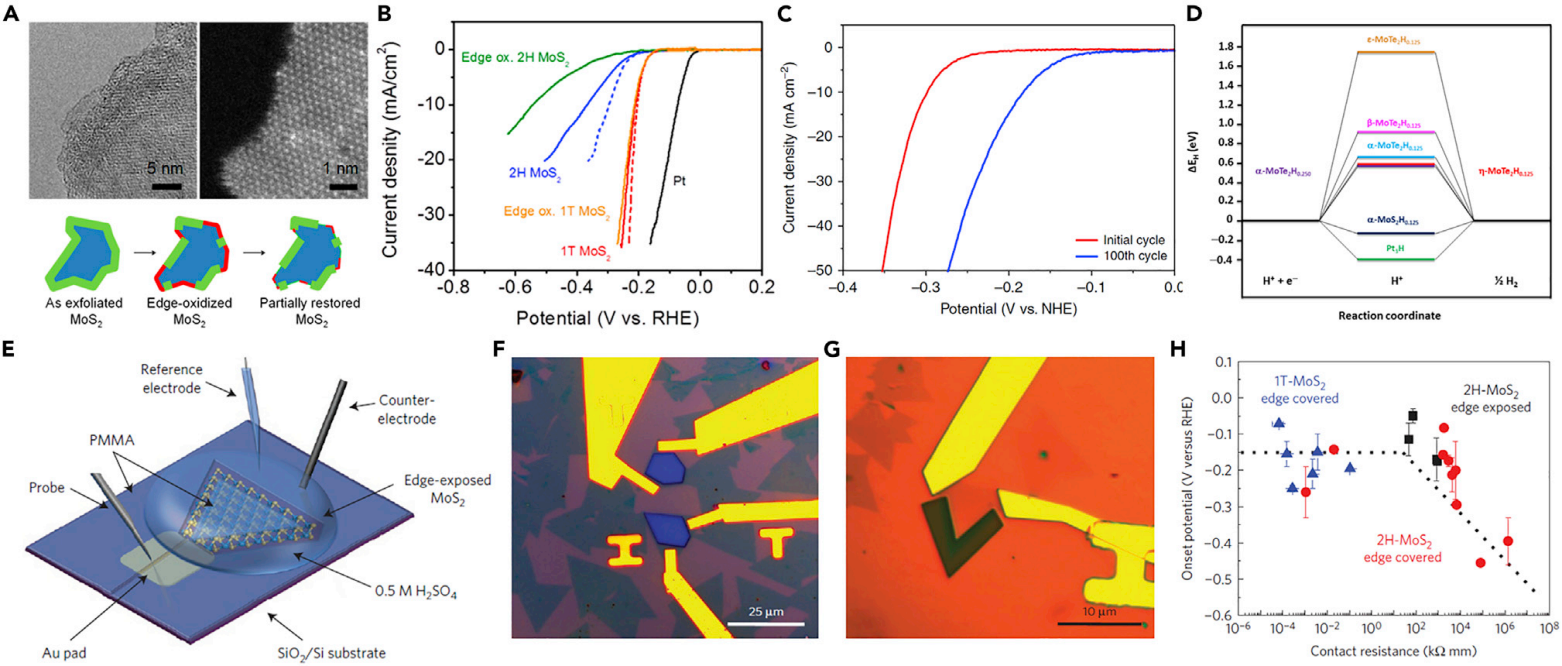
[HER intrinsic activity of group VIB MTMDs] (A and B) Confirmation of intrinsic active sites to absorb hydrogen for 1T-MoS_2_ using the edge-oxidation method. (A) TEM images of edge-oxidized MoS_2_ nanosheets with the schematic of the oxidation process and partial restoration of the edges after several cycles. (B) Polarization curves of 1T and 2H MoS_2_ nanosheet electrodes before and after edge oxidation. Reprinted with permission from (Voiry et al., 2013a). Copyright 2013, American Chemical Society. (C, D) HER performance of 1T′-MoTe_2_ derived from the fundamental electronic structure. (C) LSV curves and (D) comparison of Δ*G*_H∗_ values at various H-bonding sites. Reprinted with permission from (McGlynn et al., 2019). Copyright 2019, The Authors published by Springer Nature. (E-H) Improvement of HER intrinsic property for MoS_2_ via electrical contact engineering. (E) Schematic of electrochemical device setup on a single-layer MoS_2_. OM images of (F) edge-covered and (G) edge-exposed monolayer MoS_2_-based microcell. (H) Onset potential dependent contact resistance of 2H-MoS_2_ edge sites, 2H-MoS_2_ basal plane, and 1T-MoS_2_ basal plane. Reprinted with permission from (Voiry et al., 2016). Copyright 2016, Nature Publishing Group.

Thermodynamically stable group VIB MTMDs indicate inhibited electrochemical capabilities because of their semiconducting nature. For enhancing the original catalytic activities of group VIB MTMDs, the phase transition from the semiconducting 2H to the metallic 1T phase was demonstrated as the facile strategy (Hu et al., 2017a; Tang and Jiang, 2016). Combined theoretical and experimental methodologies reveal that the conversion of 2H to 1T MoS_2_ enhances HER catalytic performance (Lukowski et al., 2013; Wang et al., 2013). Voiry et al. proved that the highly concentrated conducting 1T phase of exfoliated MoS_2_ nanosheets induced superior HER activity via experiments (Voiry et al., 2013a). Edge sites of 2H and 1T-MoS_2_ were partially oxidized by soaking in aqueous solutions with oxygen saturation and performing cycles, which were confirmed by TEM images (Figure 8A), to investigate the catalytic mechanism. The edge-oxidized 2H-MoS_2_ presented a more decreased activity than 2H-MoS_2_, whereas the performance of 1T-MoS_2_ and edge-oxidized 1T-MoS_2_ is similar (Figure 8B). This result indicates that the oxidation of 1T-MoS_2_ is unaffected by the catalytic properties. McGlynn et al. reported that the changes of electronic structure in 1T′-MoTe_2_ dramatically improve catalytic activity when operating HER at the cathodic bias (McGlynn et al., 2019). As shown in Figure 8C, the overpotential (η) of 1T′-MoTe_2_ reduced from 320 mV to 178mVat the current density of 10mVcm^−2^ after only 100 cycles. Based on the theoretical calculation, HER activity can be enhanced by changes in the electronic structure caused by electron doping under an applied potential while maintaining 1T′-MoTe_2_ (Figure 8D).

On-chip devices have been used to investigate atomically intrinsic active sites by exposing selective surfaces and to facilitate catalytic activity by enhancing the electrical coupling of MTMDs (Yang et al., 2020; Zhang et al., 2017a). The edge contact technology using micro-electrochemical cell is essential to minimize contact resistance and confirm inherent catalytic properties (Zhou et al., 2018c). As shown in Figure 8E, a microelectrochemical cell composed of monolayer MoS_2_ was manufactured initially (Voiry et al., 2016). The HER activity according to the active site was selectively evaluated by covering or exposing the edge of MoS_2_ using e-beam lithography (Figures 8F and 8G). Figure 8H shows that HER activity at the basal plane of MoS_2_ irrespective of the phases is improved by decreasing the contact resistance and increasing the charge transfer, which demonstrates a mutual relationship between catalytic properties and electrical coupling.

### Group VB MTMDs

**Figure 9:**
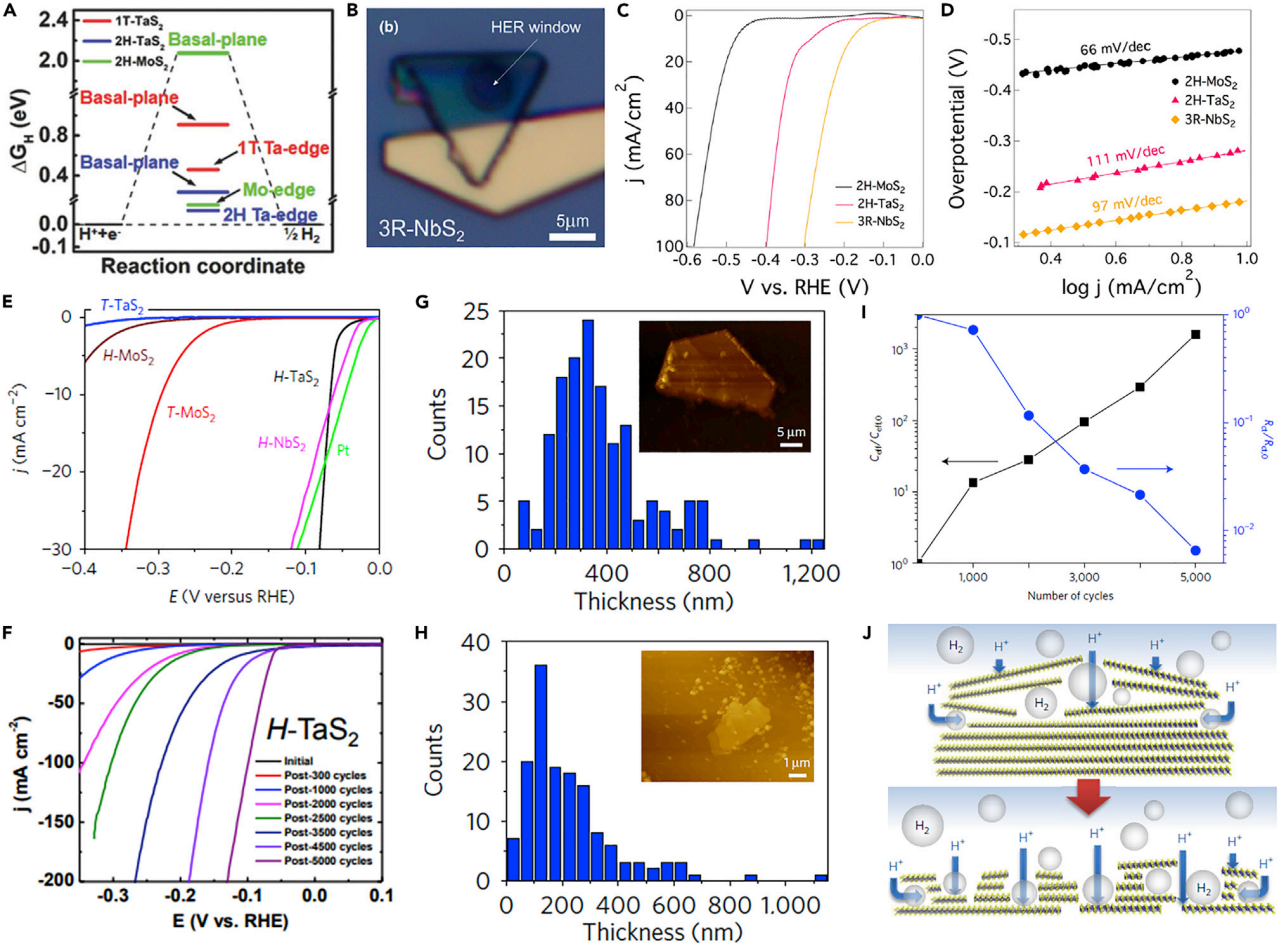
[HER intrinsic activity of group VB MTMDs] (A) Δ*G*_H∗_ diagram of the different active sites for 1T-TaS_2_, 2H-TaS_2_, and 2H-MoS_2_. Reprinted with permission from (Huan et al., 2018). Copyright 2018 WILEY-VCH Verlag GmbH & Co. KGaA, Weinheim. (B–D) Discovery of superior active basal plane for 3R-NbS_2_. (B) Basal plane exposed 3R-NbS_2_. (C) Polarization curves and (D) Tafel slopes of 3R-NbS_2_, 2H-TaS_2_, and 2H-MoS_2_. Reprinted with permission from (Zhang et al., 2019a). Copyright 2019 Elsevier Ltd. (E–J) Self-optimized catalytic activity of group VB MTMDs (*H*-TaS_2_ and *H*-NbS_2_). (E) Polarization curves of *H*-TaS_2_, *H*-MoS_2_, and *H*-NbS_2_. (F) Polarization curves performed periodically during potential cycling in *H*-TaS_2_. The thickness distribution of *H*-TaS_2_ with the AFM image (G) before (H) and after cycling. (I) Variation of effective capacitance (C_eff_/C_eff,0_) and charge-transfer resistance (R_ct_/R_ct,0_) with an increase in the cycle numbers for *H*-TaS_2_. (J) Illustration of self-optimizing processes attributed to the morphological change during cycling. Reprinted with permission from (Liu et al., 2017). Copyright 2017, Nature Publishing Group.

Although the catalytic activity of semiconducting group VIB TMDs is revealed only on edge sties, group VB MTMDs comprising diverse crystal structures exhibit highly active sites on both edges and basal planes based on theoretical DFT results for Δ*G*_H∗_ in Figure 9A (Huan et al., 2018). Zhang et al. analyzed the inherent properties of metallic 3R-NbS_2_, 2H-TaS_2_, and 2H-MoS_2_ basal planes applying electrochemical on-chip devices (Figure 9B) (Zhang et al., 2019a). The basal plane of the grown 3R-NbS_2_ flakes by a chemical solid reaction indicates an admirable activity in the HER measurement. 3R-NbS_2_ exhibited a relatively less overpotential of 182 mV to reach a current density (10mVcm^−2^) and a larger exchange current density of 1.3 × 10^−4^ A cm^−2^ from the Tafel slope than those of 2H-MoS_2_ (479 mV and 6.3 × 10^−10^ A cm^−2^) (Figures 9C and 9D). The basal plane of group VB MTMDs, especially NbS_2_, was confirmed as the active site and was considered a promising HER catalysis beyond group VIB MTMDs.

Some group VB MTMDs catalysts such as TaS_2_ and NbS_2_ have the unusual intrinsic ability of self-optimum performance based on highly enhanced active basal plane sites when negative potentials are continuously applied for HER activation (Shi et al., 2017; Zeng et al., 2014). In addition, Liu et al. addressed the HER kinetics of group VB MTMDs for H-TaS_2_ and H-NbS_2_ multilayer platelets prepared by CVD growth (Liu et al., 2017). Compared to different MTMDs-based catalysts, H-TaS_2_ requires a relatively low overpotential of 60 mV to yield a current density of 10mAcm^−2^ (Figure 9E). H-TaS_2_ needs to repeat 5,000 cycles for optimizing catalytic activity to achieve this HER performance (Figure 9F). Atomic force microscopy (AFM) profiling as shown in Figures 9G and 9H displays a thinner thickness of optimized H-TaS_2_ that ranges from about 100 nm to 150 nm than that of H-TaS_2_ measured before cycling (approximately 300 nm to 400 nm); this implies that these morphological changes acquired during electrochemical reaction result in enhanced catalytic activity. Charge-transfer resistances are obtained by the electrochemical impedance spectra (EIS) decreased following the repeated number of cycles until 5,000 cycles, which denotes shorter electron-transfer pathways. The calculated double-layer capacitance by EIS analysis boosted as cycling measurement is repeated, and this implies an increase in the active surface area (Figure 9I). Following these beneficial features, they proposed that the generated H_2_ bubbles on the basal plane between group VB MTMDs interlayers can be trapped; the trapped H_2_ gas moves to escape leading to exfoliate or perforate layers (Figure 9J). The self-optimizing HER activity of the pre-mentioned group VB MTMDs (H-TaS_2_ and H-NbS_2_) improved the charge transfer and accessibility of active sites induced by morphological changes. This offers a promising platform to apply scalable electrochemical devices that can surpass traditional TMDs.

### Defect engineering

**Figure 10:**
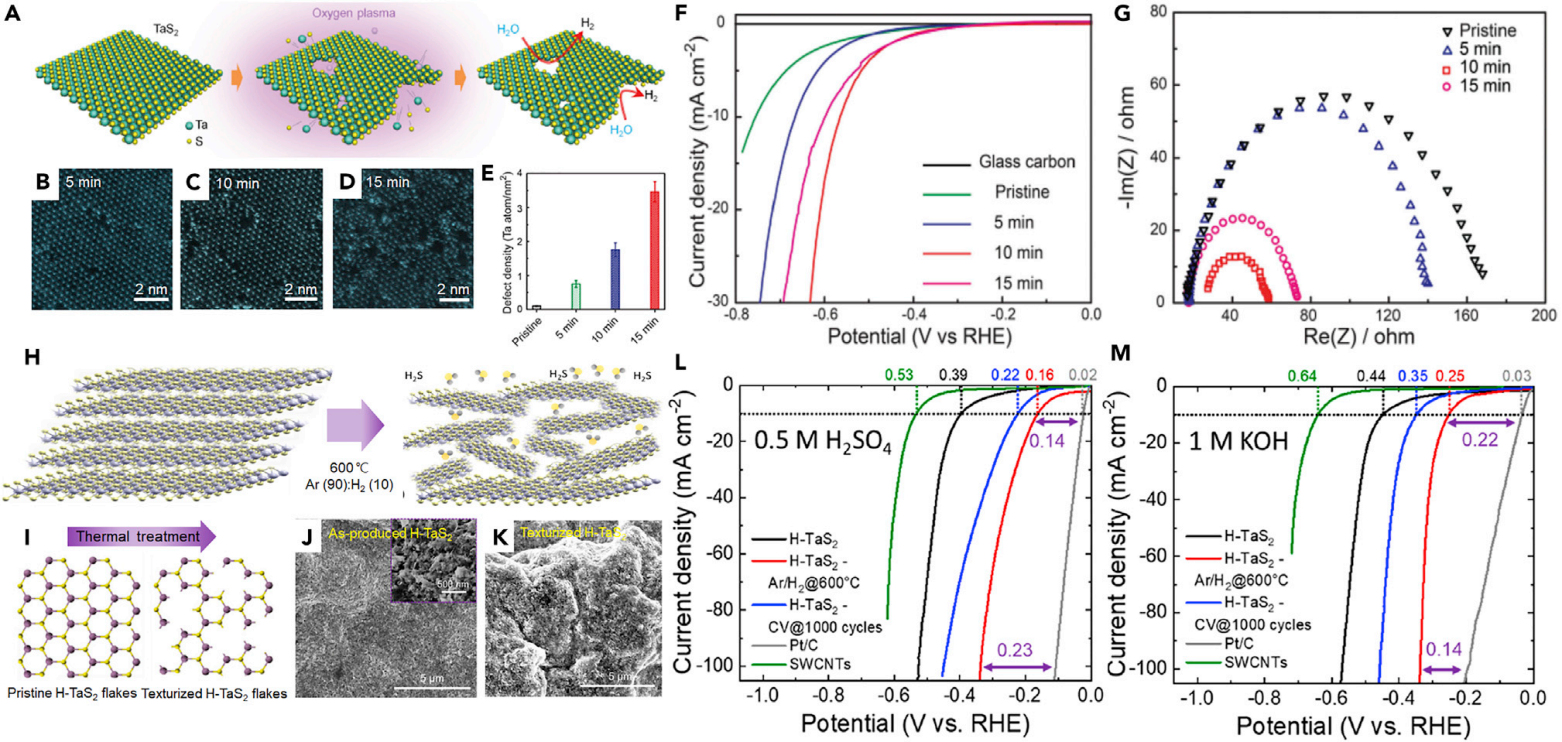
[Post-treatment for defect engineering of MTMDs to enhance HER activity] (A–G) Development of the defective basal plane of TaS_2_ with atomic-sized pores for activating catalysts. (A) Schematic of O_2_ plasma treatment of TaS_2_ nanosheets with atomic-sized pores. TEM images of plasma-operated TaS_2_ flakes for (B) 5 min, (C) 10 min, and (D) 15 min. (E) The increment of Ta defect density during the O_2_ plasma treatment. (F) Polarization curves and (G) Nyquist plots measured from various plasma operating times. Reprinted with permission from (Li et al., 2016a). Copyright 2016 WILEY-VCH Verlag GmbH & Co. KGaA, Weinheim. (H–M) Effect of defect treatment on the HER performance of the TaS_2_ catalyst. (H) Schematic of the texturization of the H-TaS_2_ flakes. (I) Illustration of the number of Ta edge sites in H-TaS_2_ enriched during a thermal reaction. SEM images of the H-TaS_2_ films (J) before (inset: high-resolution image) and (K) after annealing. LSV curves of synthesized catalysts (L) in 0.5 M H_2_SO_4_ and (M) 1.0 M KOH electrolyte, respectively. Reprinted with permission from (Najafi et al., 2020). Copyright 2020, American Chemical Society.

Numerous reports on experimental and theoretical studies have established that increasing the density of edge sites leads to the enhancement of catalytic activity in the TMDs materials (Lin et al., 2016; Noh et al., 2018; Sarma et al., 2019). The facile process for creating defects that transform the electronic structure has been developed to increase edge sites (Hong et al., 2015; Li et al., 2018c). Post-treatments performed after the growth of materials using strain (Li et al., 2016b; Voiry et al., 2013b), thermal annealing (Najafi et al., 2020; Yin et al. 2016), and plasma (Ye et al., 2016) provide controllable defect density and improve active edge sites. Figure 10A shows low-energy oxygen (O_2_) plasma processing induced a tremendous density of atomic-scale pore defects in the basal plane of metallic TaS_2_ sheets (Li et al., 2016a). The result of STEM analysis (Figures 10B–10E) shows the number of pores controlled depending on the plasma treatment time from 0 to 15 min. Treated TaS_2_ electrocatalysts with an optimal defect concentration for 10 min showed the lowest onset potential of 200 mV and charge transfer resistance (Figures 10F and 10G). The post-treatment using O_2_ plasma that generates a large number of atomic-scale pores was demonstrated to increase the exposed edge of TaS_2_ in the basal plane, which enhances HER activity. In addition, Najafi et al. presented thermal annealing under an H_2_-rich atmosphere as post-treatment to control the defect of metallic TaS_2_ (Najafi et al., 2020). The TaS_2_ films obtained through the filtration of the colloidal solution comprising 2H-TaS_2_ flakes were annealed at 600°C under Ar/H_2_. Hydrogen plays a major role in TaS_2_ etching while generating H_2_S gas (Figure 10H). The annealed 2H-TaS_2_ films exhibited boosted porosity for promoting the ion adsorption rate and increased quantity of edge sites because of diminishing sulfur content (∼14%) (Figures 10I–10K). These properties of the 2H-TaS_2_ electrode significantly influence catalytic activity in both acidic and basic electrolytes. Compared with the conventionally obtained 2H-TaS_2_ after electrochemical 1000 cycles, annealed 2H-TaS_2_ catalyst displayed relatively low overpotential under 0.5 M H_2_SO_4_ (160 mV) and 1.0 M KOH (250 mV) solutions, respectively (Figures 10L and 10M). The subsequently thermal treatment is considered suitable engineering by increasing its porosity and catalytic active sites to accelerate the electrochemical reaction of the prepared TaS_2_. In addition to plasma and thermal annealing, strain (Li et al., 2016b) is another important method that can modulate the defects influencing the HER intrinsic catalytic activity. Locally strained lattices in the zigzag chain reduce the energy required for phase transformation (Voiry et al., 2013b) and thus modulate the hydrogen adsorption and desorption (Li et al., 2016b; Putungan et al., 2015). Voiry et al. reported a stable, strained 1T-WS_2_ with high HER activity. As the introduction of tensile strain (∼3%) leads to an enhancement in the density of states near the Fermi level, their Δ*G*_H∗_ approaches zero. In contrast, the compressive strain would cause Δ*G*_H∗_ on MTMDs (*e*.*g*., 1T-MoS_2_ and 1T-NbS_2_) to move further away from zero and decrease the HER activity (Chen and Wang et al., 2016).

**Figure 11:**
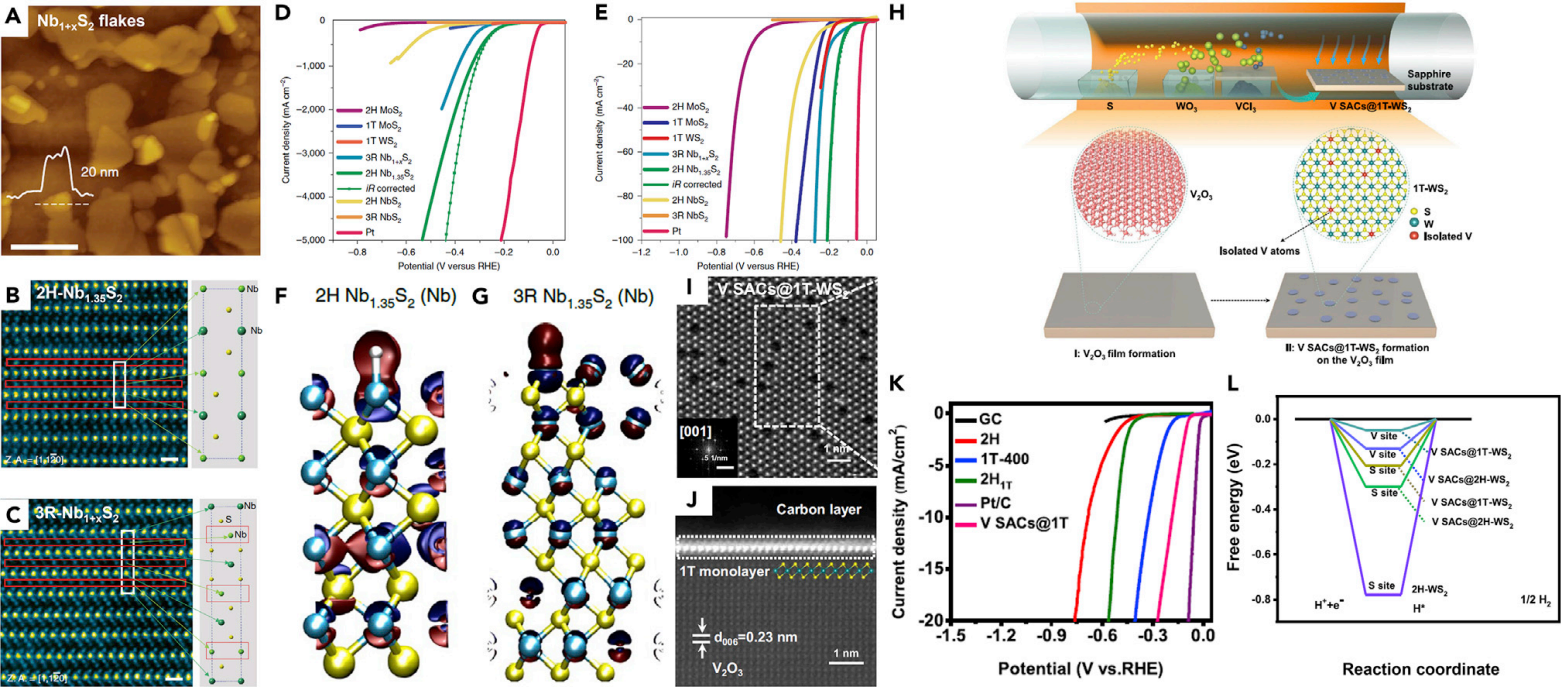
[Controllable defects during the growth of MTMDs] (A-G) Interstitial defect engineering of Nb_1+*x*_S_2_ to enhance the catalytic performance. (A) AFM image of Nb_1+*x*_S_2_. Cross-section ADF STEM images of (B) 2H-Nb_1.35_S_2_ and (C) 3R-Nb_1+*x*_S_2_ with a unit cell of structures. (D) Polarization and (E) expanded curves under the low potential from (D) of 2H-Nb_1.35_S_2_ with the other TMD-based catalysts. Charge density distribution for H adsorbed on the Nb-terminated (F) 2H-Nb_1.35_S_2_ and (G) 3R-Nb_1+*x*_S_2_. Reprinted with permission from (Yang et al., 2019). Copyright 2019, The Authors, under exclusive licence to Springer Nature Limited. (H–L) V single atomic doping in 1T-WS_2_ toward HER catalyst. (H) Schematic of the one-step synthetic method for V SACs@1T-WS_2_ monolayer. (I) HR HAADF-STEM images of V SACs@1T-WS_2_. Inset: fast Fourier transform (FFT) image. (J) Cross-section image of HR HAADF-STEM for V SACs@1T-WS_2_ on the V_2_O_3_ substrate. (K) The LSV curves of as-grown catalyst with different counterparts. (L) Comparison of Δ*G*_H∗_ values for diverse sties. Reprinted with permission from (Han et al., 2021). Copyright 2021, The Authors published by Springer Nature.

Another strategy to introduce defect sites in the MTMDs includes the control of synthetic parameters such as precursor and growth conditions. Studies on the correlation between catalytic properties and atomic vacancy have been reported. He et al. showed that the 1T-MoS_2_ content can be easily controlled by varying the pyrolysis temperature or Mo/S feeding molar ratios (He et al., 2019). The disordered stacking of S-Mo-S layers and the abundant defects formed during pyrolysis are synergistically responsible for their high HER activity. However, the content of defective sites in MTMDs must be considered for optimizing the conductivity of the catalysts because the atomic vacancies eventually deteriorate the electrical properties. Recently, the scope of defects has been expanded to a strategy that can simultaneously improve electrical and catalytic characteristics. Yang et al. utilized a covalently bonded MTMDs by self-intercalation (Yang et al., 2019). The metallic 2H and 3R NbS_2_ crystals with excess niobium (2H-Nb_1+*x*_S_2_, 3R-Nb_1+*x*_S_2_, and where *x* is ∼0.35) are synthesized via the adjustment of CVD synthesis parameters. The prepared Nb_1.35_S_2_ materials contained variable thicknesses (2∼50 nm) and lateral flake size (0.5∼1 μm) determined by the AFM image (Figure 11A) wherein the crystal structures were identified as excess Nb on both the 2H and 3R-Nb_1.35_S_2_ phase from the ADF-STEM images (Figures 11B and 11C). During the partial occupation of the surplus Nb between 2D metallic NbS_2_ layers, the influence of van der Waals forces in the 2D layers declines, which induces great abilities of fast charge transfer and high current capability. Therefore, the 2H-Nb_1.35_S_2_ for HER generated an ultrahigh current density of 5,000mAcm^−2^ at an overpotential of 420 mV (Figures 11D and 11E). The charge density for hydrogen adsorption onto the Nb-terminated surface was theoretically calculated to demonstrate the intrinsic mechanism of Nb_1.35_S_2_ catalysts (Figures 11F and 11G). These results suggest that the localized charge density of the 2H-Nb_1.35_S_2_ phase is larger in magnitude than that of the 3R-Nb_1.35_S_2_ phase. The Nb self-intercalation in 2H-NbS_2_ fabricated by controlling the growth condition of the pressure provides facile defect design to advance HER activity. The chemical doping of MTMDs using various metal dopants such as Co, Ni, Cr, V, or Re can tune the electronic structure (He et al., 2020). Han et al. presented a one-step CVD method as the facile doping strategy for synthesizing a V single atom-doped metallic 1T WS_2_ (V SACs@1T-WS_2_) monolayer (Han et al., 2021). The V SACs@1T-WS_2_ was grown using tungsten trioxide and sulfur while introducing vanadium chloride as the co-precursor (Figure 11H). Figure 11I shows the atomic structure of high-resolution HAADF-STEM images for V SACs@1T-WS_2_ monolayer, which indicates that W atoms are replaced by V atoms. The cross-section HAADF-STEM image in Figure 11J indicates the epitaxial bonding between the V SACs@1T-WS_2_ and V_2_O_3_ substates in a monolayer, which implies the high-metallic 1T-phase purity of 91%. The catalytic properties of V SACs@1T-WS_2_ exhibit lower ᶯ_10_ (∼185 mV) comparable to 2H-WS_2_ counterparts (Figure 11K). The various types of active site in the V single-atomic doped WS_2_ catalysts were scrutinized via the theoretical calculation of Δ*G*_H∗_. The V-atom sites in the 1T-WS_2_ monolayer shows the lowest value of Δ*G*_H∗_ (0.05 eV) closed to zero, which reveals a superior intrinsic catalytic activity (Figure 11L). Accordingly, V single-atomic doping considerably affected enhancing the electrocatalytic ability of intrinsic 1T-WS_2_ single-layer.

### Interfacial engineering

**Figure 12:**
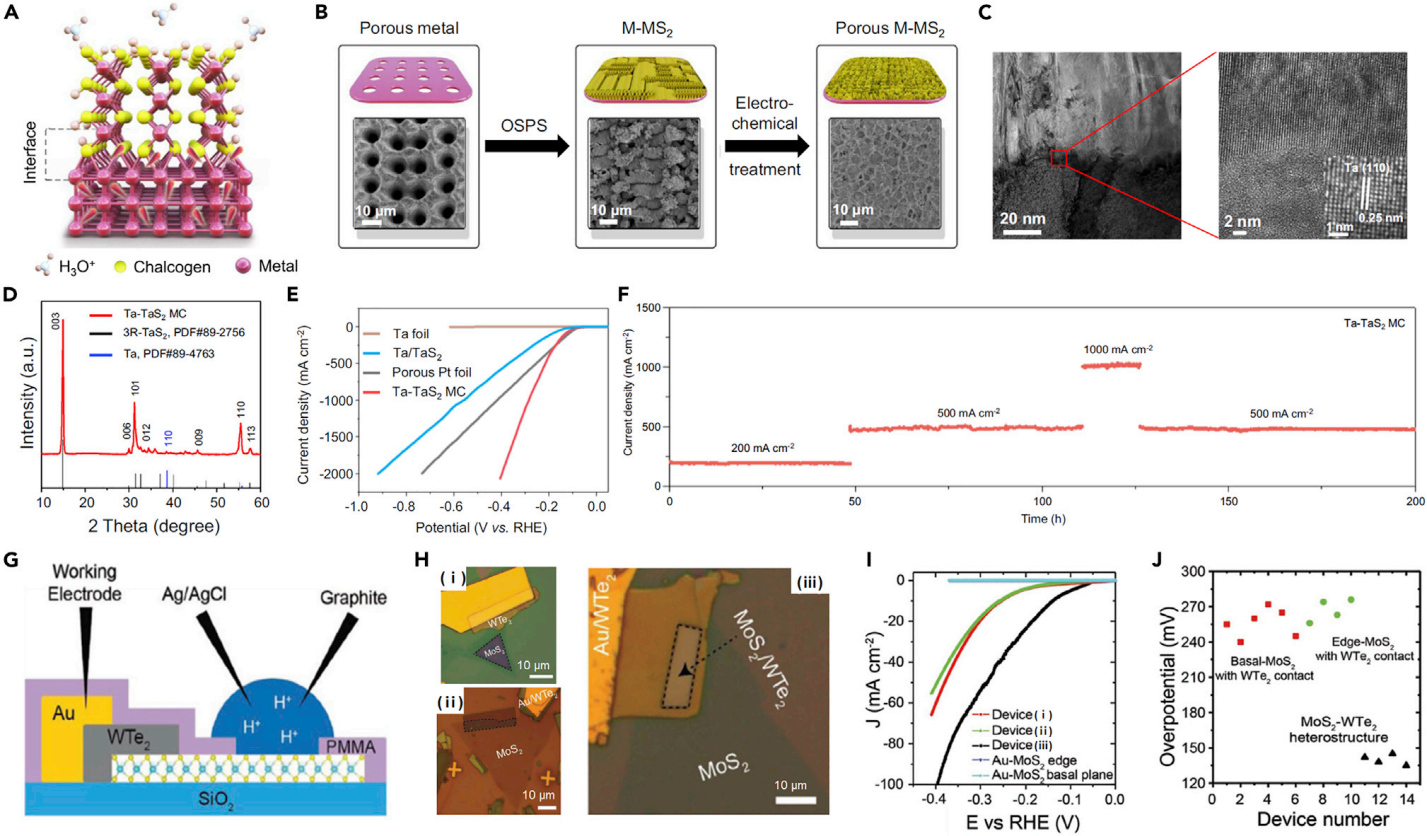
[Interfacial engineering of MTMDs for enhancing HER activity] (A–F) Advancement of TaS_2_ catalyst using facile metallic interface. (A) Illustration of atomic structure for the Ta-TaS_2_ MC. (B) Synthesis procedure of porous Ta-TaS_2_ MC with SEM images. (C) Cross-section TEM images of Ta-TaS_2_ interface. (D) XRD patterns of Ta-TaS_2_ MC. (E) Polarization curves of Ta-TaS_2_ MC compared with Ta foil, porous Pt foil, and Ta/TaS_2_. (F) Chronoamperometry of Ta-TaS_2_ MC measured at various current densities for 200 h. Copyright 2021 Springer Nature. Reprinted with permission from (Yu et al., 2021). Copyright 2021, The Authors published by Springer Nature. (G–J) Enhanced original property toward HER of MoS_2_ by interfacial effects with metallic WTe_2_. (G) Cross-section schematic of the MoS_2_-WTe_2_ heterostructure HER microreactor. (H) OM images of (i) basal plane exposed, (ii) edge exposed WTe_2_ contacted MoS_2_, and (iii) basal plane exposed MoS_2_-WTe_2_ heterostructures. (I) Polarization curves of each device. (J) Comparison of overpotential values in different devices from polarization curves. Reprinted with permission from (Zhou et al., 2019). Copyright 2019 WILEY-VCH Verlag GmbH & Co. KGaA, Weinheim.

Most catalytic activity of MTMDs for applying electrochemical systems has been measured through catalysts deposited on conductive electrodes (such as carbon paper, glassy carbon, and Ni foam) with binders such as Nafion or polyvinylidene fluoride (PVDF) (Yi et al., 2021; Yu et al., 2014; Zhang et al., 2019c). These additives have critical issues that restrict electrochemical reaction and increases interface resistance because of their inactive and insulating characteristics (Zhang et al., 2019b). Binder-free electrodes have been considered practical catalysts improving adhesion energy between materials and substrates with small interface resistance to overcome this limitation (Hu et al., 2017b; Liu et al., 2018a). Yu et al. found that MTMDs directly grown on a substrate of the equal metal were synthesized by utilizing oriented-solid-phase synthesis (OSPS) to facilitate the mobility of charge injection in the catalysts (Figure 12A) (Yu et al., 2021). They suggested novel systems to fabricate monolith catalyst (MC) based on metallic TaS_2_ vertically grown onto the Ta metal (Ta-TaS_2_ MC). Figure 12B shows the synthesis of porous Ta-TaS_2_ by electrochemical treatment. The cross-section TEM images in Figure 12C clearly reveal an abrupt interface between Ta metal and TaS_2_ with intensive covalent bonds. As shown in XRD pattern (Figure 12D), the structure of the as-synthesized TaS_2_ is 3R-phase. Ta-TaS_2_MC accomplished a superior current density of 2,000mAcm^−2^ with a low overpotential of 398 mV and excellent durability for 200 h toward the commercialization of hydrogen production when the Ta-TaS_2_ MC examined HER activity compared with porous Pt foil, Ta foil, and conventional parallel Ta/TaS_2_ (Figures 12E and 12F). These impressive performances of Ta-TaS_2_ MC catalysts rose from its features for mechanical strength and electrically near-zero interface resistance. Furthermore, they contributed to the emerging importance of delicate interfacial research for industrializing water electrolyzers. Zhou et al. proposed the method using charge injection between 2H-MoS_2_ and T_d_-WTe_2_ via band engineering to improve the interfacial properties (Figure 12G) (Zhou et al., 2019). Three devices composed of T_d_-WTe_2_ and 2H-MoS_2_ were fabricated corresponding to: (1) Basal plane-MoS_2_ contacted WTe_2_, (2) edge-MoS_2_ contacted WTe_2_, and (3) basal plane of MoS_2_-WTe_2_ heterostructure to verify catalytic activity for the selective windows of MoS_2_ (Figure 12H). The engineered heterostructure (device (iii) in Figure 12H) exhibited the ultimate HER performance (η at 10mA cm^−2^ (η_10_) ≈ 150 mV) than WTe_2_ contacted MoS_2_ (device (i, ii), η_10_ ≈ 255 mV), as shown in Figures 12I and 12J. The microdevice of the monolayer MoS_2_ demonstrated that the heterojunction between MoS_2_ and metallic WTe_2_ affect the enhancement of the catalytic characteristic attributed to efficient charge injection.

### Other approaches of structural engineering

**Figure 13:**
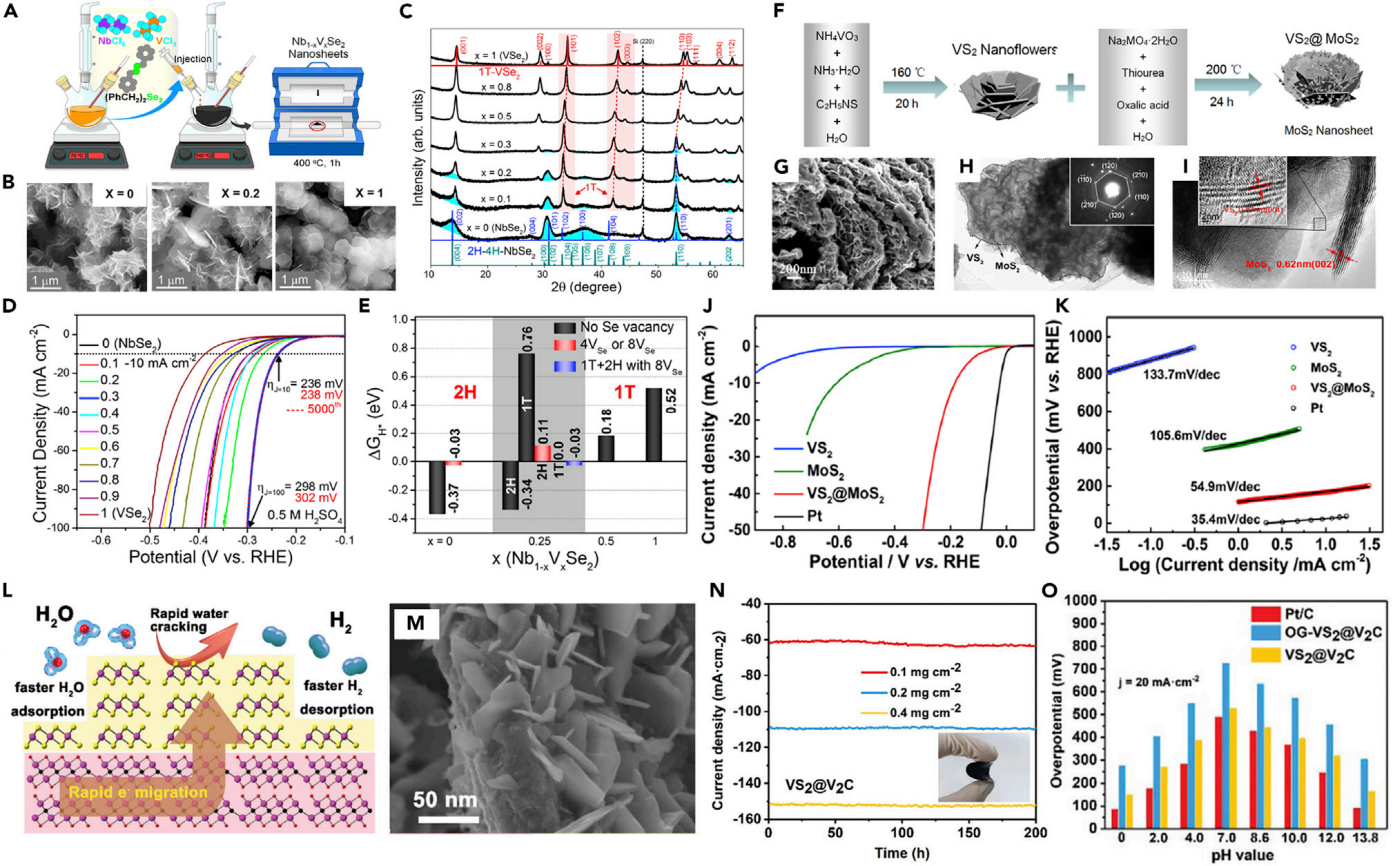
[Diverse structure engineering of MTMDs] (A–E) Affect alloy control of Nb_1-*x*_V_*x*_Se_2_ on the structural phase and HER performance. (A) Scheme of synthesis for Nb_1-*x*_V_*x*_Se_2_ using the aqueous colloidal method. (B) SEM images of the Nb_1-*x*_V_*x*_Se_2_ at *x* = 0, 0.2, 1. (C) XRD patterns of various Nb_1-*x*_V_*x*_Se_2_. (D) HER LSV curves and (E) calculated Δ*G*_H∗_ values of Nb_1-*x*_V_*x*_Se_2_ Reprinted with permission from (Kwak et al., 2022). Copyright 2022 American Chemical Society. (F–K) Enhanced HER activity of 1T-VS_2_ through heterojunction with MoS_2_. (F) Schematic showing the fabrication of the VS_2_@MoS_2_ by two-step hydrothermal reaction. (G) SEM, (H) TEM with SAED pattern, and (I) HRTEM image of VS_2_@MoS_2_. (J) HER polarization curves and (K) Tafel slopes of VS_2_@MoS_2_ with different catalysts. Reprinted with permission from (Chen et al., 2017). Copyright 2017 American Chemical Society. (L–O) HER activity of the composite 1T-VS_2_ with V_2_C MXene. (L) Illustration of hydrogen evolution process in VS_2_@V_2_C structure. (M) SEM image of VS_2_@V_2_C. (N) Overpotential comparison at full pH value and (O) chronoamperometry of the fabricated VS_2_@V_2_C films in seawater. Reprinted with permission from (Wang et al., 2020). Copyright 2020 RSC Pub.

A beneficial structural design such as alloy (Huang et al., 2019a; Kwak et al., 2020; Kwon et al., 2021) and hybrid structures (Gnanasekar et al., 2020; Zhou et al., 2018b) have been recently dedicated to magnifying the electrochemical capability of MTMDs. Alloying corresponding to the stoichiometry modification of MTMDs compounds mediates conductivity and electronic structure by affecting catalytic activities (Xu et al., 2017). Kwak et al. proposed the polytype alloys of Nb_1-*x*_V_*x*_Se_2_ with the metallic nature as the efficient HER catalysts (Kwak et al., 2022). The Nb_1-*x*_V_*x*_Se_2_ nanosheets under all composition ranges were grown by a hot-injection colloidal reaction and annealing process (Figure 13A). The typical SEM images of the Nb_1-*x*_V_*x*_Se_2_ alloys as a function of composition indicates that the morphologies transformed from nanosheets to thick nanoplates as *x* increased from 0.2 to 1.0 (Figure 13B). The Nb_1-*x*_V_*x*_Se_2_ alloys have a crystal structure of a combination of 2H and 1T phases when *x* is in the range of 0.1 to 0.3 and have a 1T-phase when *x* is relatively high (Figure 13C). The as-prepared Nb_1-*x*_V_*x*_Se_2_ nanosheets were examined for electrochemical activities; the Nb_0.7_V_3_Se_2_ (*x* = 0.3) showed the ultimate HER performance, which demonstrates that η is the lowest value 236 and 298mVat a current density 10 and 100mAcm^−2^, respectively (Figure 13D). From the Δ*G*_H∗_ of Nb_1-*x*_V_*x*_Se_2_ alloys as a function of composition; they confirmed thermoneutral at *x* = 0.3 and proved the interrelation of the modification for alloy composition with enhanced HER activity (Figure 13E).

The hybridization between STMDs and MTMDs was reported to enhance fundamental charge transfer efficiency and electrochemical long-term stability (Du et al., 2018). Chen et al. manufactured the MoS_2_ nanosheets with metallic 1T-VS_2_ (VS_2_@MoS_2_) via two-step hydrothermal reactions (Figure 13F) (Chen et al., 2017). Representative SEM and TEM images illustrate the distinct formation of MoS_2_ nanosheets on the surface of VS_2_ nanoflowers (Figures 13G–13I). The VS_2_@MoS_2_ heterostructure with a low η_10_ (∼177 mV) and Tafel slope (∼54.9 mV dec^−1^) demonstrated a higher HER activity than those of the pristine VS_2_ and MoS_2_ (Figures 13J and 13K). Consequently, they suggested VS_2_@MoS_2_ heterostructure as electrocatalysts for HER to realize developed electrochemical systems. As a further strategy, composites of MTMDs and conductive materials have been used to improve the catalytic activity and stability. This approach exploits the synergistic benefits of the high catalytic activity of MTMDs, along with the controllable surface, high conductivity, and stable electrochemical properties of the conductive supports. Wang et al. suggested that the HER performance of 1T-VS_2_ can be improved by compounding it with V_2_C MXene (Wang et al., 2020). When 1T-VS_2_ with V_2_C MXene was synthesized via a hydrothermal reaction, the outstanding properties of MXene, such as large surface area and high electrical conductivity, accelerated electron charge transfer, increased the number of exposed HER active sties, and prevented the aggregation of 1T-VS_2_ (Figures 13L and 13M). The VS_2_@V_2_C compound exhibits not only a low *η*_10_ value (94 mV) in 0.5 M H_2_SO_4_, but also better activity comparable to Pt/C under a wide range of pH conditions (Figure 13N). They provided a practical design of compositional structures to achieve superior performance by demonstrating the excellent stability of VS_2_@V_2_C for 200 h (Figure 13O).

## Challenges and perspectives

Researchers continue to focus on developing efficient and sustainable electrocatalysts for promoting the HER to achieve net-zero carbon emission as the world faces an energy crisis. Among some advanced non-precious-based electrocatalysts, MTMD materials provide the most extensive prospects for material design because of eco-friendly property, ultrahigh conductivity, and tunable and abundant catalytic activity sites. This review summarized extraordinary characteristics and different advances of MTMDs considering synthetic methods and electrochemical catalytic applications (Tables 3 and 4). However, several challenges with current techniques to fulfill the industrialization of MTMD-based electrocatalysts need to be addressed. We hope that the following perspectives will assist the research community on MTMDs materials to surpass current advances in the emerging field of electrocatalysts based on MTMDs and motivate them to develop practical applications ranging from catalytic to electronics and optoelectronics.

**Table 3:** Summary of the fabrication and application of group VIB MTMDs-based electrocatalysts

Fabrication			Strategy for HER	Electrode		Overpotential		Tafel slope (mV dec^−1^)	Stability (Cycle, CV), electrolyte	Ref.
Approach	Synthesis	Template or precursor		Catalyst	Substrate (Direct growth or transfer)	Onset (η_onset_)	at 10mAcm^−2^ (η_10_)			
Top-down	Chemical exfoliation using Li intercalation	Bulk 2H-MoS_2_ grown by CVT	Phase transition	1T-MoS_2_	Glassy carbon (Transfer)	N/A	200	40	N/A in 0.5 M H_2_SO_4_	(Voiry et al., 2013a)
	Chemical exfoliation using Li intercalation	Bulk 2H-WS_2_ powder	Phase transition	1T-WS_2_	Glassy carbon (Transfer)	100	235	60	>10,000 for 120 h in 0.5 M H_2_SO_4_	(Voiry et al., 2013b)
	Chemical exfoliation using Li intercalation	Bulk 2H-MoS_2_ powder	Phase transition, defect engineering using annealing	Mesoporous 1T-MoS_2_	Glassy carbon (Transfer)	N/A	153	43	1,000 in 0.5 M H_2_SO_4_	(Yin et al., 2016)
	Ball milling and chemical Li intercalation	Bulk 2H-MoSSe grown by CVT	Phase transition, alloying	1T-MoSSe	Glassy carbon (Transfer)	49	140	40	10,000 in 0.5 M H_2_SO_4_	(Tan et al., 2018)
Bottom-up	Chemical Li intercalation	2H-MoS_2_ nanosheet grown by CVD	Phase transition	1T-MoS_2_	Si/SiO_2_ (Direct growth)	150	N/A	65	N/A, in 0.5 M H_2_SO_4_	(Voiry et al., 2016)
	CVD	K_2_MoS_4_ powder	–	1T′-MoS_2_	Graphite (Direct growth)	205	N/A	51	1,000 in 0.5 M H_2_SO_4_	(Liu et al., 2018b)
	CVD	WO_3_, VCl_3_, and S powder	Doping	Single atom-V doped 1T-WS_2_	Glassy carbon (Transfer)	N/A	185	61	2,000 in 0.5 M H_2_SO_4_	(Han et al., 2021)
	CVD	W film, Te powder	Interfacial engineering	2H-MoS_2_/T_d_-WTe_2_ heterostructure	Carbon fiber (Transfer)	50	140	40	3,000 in 0.5 M H_2_SO_4_	(Zhou et al., 2019)
	Solvothermal	(NH_4_)_2_MoSO_4_								
	Hydrothermal using NaBH_4_	Na_2_MoO_4_·2H_2_O, Se powder	Phase and disorder engineering	1T-MoSe_2_	Glassy carbon (Transfer)	N/A	152	52	1,000 in 0.5 M H_2_SO_4_	(Yin et al., 2017)
	Hydrothermal using N_2_H_4_·H_2_O	Na_2_MoO_4_·2H_2_O, NaReO_4_, S powder	Alloying	Re_0.5_Mo_0.5_S_2_	Glassy carbon (Transfer)	N/A	98	54	N/A in 0.5 M H_2_SO_4_	(Kwak et al., 2020)
	Solid state synthesis	Mo and Te powder	Tuning electronic structure using electrochemical reaction	1T-MoTe_2_	Glassy carbon (Transfer)	N/A	178	68	1,000 in 1.0 M H_2_SO_4_	(McGlynn et al., 2019)

**Table 4:** Summary of the fabrication and application of group VB MTMDs-based electrocatalysts

Fabrication			Strategy for HER	Electrode		Overpotential		Tafel slope (mV dec^−1^)	Stability (Cycle, CV), electrolyte	Ref.
Approach	Synthesis	Precursor		Catalyst	Substrate (Direct growth or transfer)	Onset (η_onset_)	at 10mAcm^−2^ (η_10_)			
Top-down	Mechanical exfoliation	Bulk 1T-VSe_2_	Tuning electronic structure using gate voltage	1T-VSe_2_	Si/SiO_2_ (Transfer)	N/A	70	59	N/A, in 0.5 M H_2_SO_4_	(Yan et al., 2017)
	Mechanical exfoliation	3R-NbS_2_ grown by chemical solid reaction	Self-optimizing	3R-NbS_2_	Si/SiO_2_ (Transfer)	N/A	188	99	10,000 in 0.5 M H_2_SO_4_	(Zhang et al., 2019a)
	Liquid phase exfoliation	Bulk 2H-TaS_2_	Defect engineering using thermal annealing	2H-TaS_2_	SWCNT film (Transfer)	N/A	160	N/A	N/A, in 0.5 M H_2_SO_4_	(Najafi et al., 2020)
	Liquid phase exfoliation	Bulk 1T-TaS_2_	Defect engineering using O_2_ plasma	Holey 1T-TaS_2_	Glassy carbon (Transfer)	200	564	135	for 10 h in 0.5 M H_2_SO_4_	(Li et al., 2016a)
	Exfoliation by sonication	Bulk 1T-VSSe grown by CVT	Alloying	1T-VSSe	Glassy carbon (Transfer)	N/A	180	87	5,000 for 20 h in 0.5 M H_2_SO_4_	(Hu et al., 2019)
Bottom-up	Hydrothermal reaction	Na_3_VO_4_·10H_2_O, C_3_H_5_NS	Interfacial engineering	1T-VS_2_	Carbon paper (Direct growth)	N/A	42	36	2,000 for 12 h in 0.5 M H_2_SO_4_	(Liang et al., 2016)
	Hydrothermal reaction	NH_4_VO_3_, C_2_H_5_NS	Heterostructure	2H-MoS_2_ on1T-VS_2_	Glassy carbon (Transfer)	97	177	54.9	1,000 in 0.5 M H_2_SO_4_	(Chen et al., 2017)
		Na_2_MoO_4_·2H_2_O, CH_4_N_2_S								
	Colloidal reaction	NbCl_5_, VCl_3_, (PhCH_2_)_2_Se_2_	Alloying	1T and 2H-Nb_0.7_V_0.3_Se_2_	Glassy carbon (Transfer)	N/A	236	72	for 24h in 0.5 M H_2_SO_4_	(Kwak et al., 2022)
	CVD using NaCl templates	TaCl_5_, S powder	Interfacial engineering via annealing	1T-TaS_2_	Au foil (Transfer)	N/A	190	61	1,000 in 0.5 M H_2_SO_4_	(Huan et al., 2019)
	CVD	TaCl_5_, S powder	Self-optimizing	2H-TaS_2_	Glassy carbon (Transfer)	N/A	60	37	N/A in 0.5 M H_2_SO_4_	(Liu et al., 2017)
		NbCl_5_, S powder		2H-NbS_2_		N/A	50	30	N/A in 0.5 M H_2_SO_4_	
	CVD	TaCl_5_, S powder	Tuning electronic structure using thickness control	2H-TaS_2_	Au foil (Direct growth)	N/A	65	33-42	N/A in 0.5 M H_2_SO_4_	(Shi et al., 2017)
	CVD	NbCl_5_, S powder	Defect engineering using Nb intercalation	2H-Nb_1.35_S_2_	Glassy carbon (Direct growth)	N/A	123	38	for 120 in 0.5 M H_2_SO_4_ h	(Yang et al., 2019)
	APCVD	TaCl_5_, S powder	Interfacial engineering	1T-TaS_2_	Nanoporous gold foam (Direct growth)	N/A	221	75	1,000 in 0.5 M H_2_SO_4_	(Huan et al., 2018)

### Reproducibility and scalable production of MTMDs materials

#### Synthetic possibility of group VIB MTMDs

A familiar synthetic method of group VIB MTMDs is the phase transition from stable 2H to metastable 1T structure by a rather complex post process such as carrier injection (Voiry et al., 2013b) and laser irradiation (Cho et al., 2015). The crystalline quality, stability, and physical properties of group VIB MTMDs can be affected by a variable factor including precursor, synthetic atmosphere, and optimization for post-treatment. Although another synthetic strategy, a solution-based approach can achieve the growth of 1T/2H-group VIB TMDs mixed phase (Zhou et al., 2021) and fully covered 1T phase via transition metal dopant (Han et al., 2021) or alloying (Kwak et al., 2022); the process parameters are difficult to control. Considering these factors, it is difficult to achieve group VIB MTMDs with reproducible results. Liu et al. recently demonstrated the use of a simple one-step synthetic process to directly grow group VIB MTMDs with the 1T′ phase via the potassium-assisted CVD (Liu et al., 2018b). This method, however, only produced a few small flakes (generally less than 1 μm), which may limit the fabrication of catalysts. Thus, more approaches with reproducibility should be explored from the perspective of the controllable and scalable production of the stable catalysts of VIB MTMDs with high quality. In recent years, Lai et al. prepared metastable 1T′-group VIB MTMDs (including WS_2_, WSe_2_, MoS_2_, MoSe_2_, WS_2x_Se_2(1-x)_, and MoS_2x_Se_2(1-x)_) using a potassium-incorporated metal precursor (Lai et al., 2021). It is worth noting that 1T′-group VIB MTMDs are thermally stable up to ∼120 °C or 160 °C and have lateral sizes up to a few hundred micrometers. Potassium-containing metal precursors (e.g., K_2_MoO_4_ and K_2_WO_4_) (Lai et al., 2021) or-metal salts (*e*.*g*., K_2_C_2_O_4_·H_2_O and K_2_CO_3_) (Lai et al., 2022) lower the energy required for TMD phase transformation, facilitate electron transfer, and inhibit electron emission. It is expected that these materials will play an important role in solving the large-area fabrication of metastable group VIB MTMDs.

### Controllable synthesis of group VB MTMDs

Unlike group VIB TMDs, the synthesis of group VB MTMDs are still in its infancy because of the limitation on the selectivity of the precursor. The low solubility of most transition metal precursors comprising group VB, as summarized in Table 2, interferes with suitable solution-based synthesis. Thus, many researchers deliberately selected the CVD method using the vaporization of a solid precursor as an optimal approach for producing group VB MTMDs materials at the atomic level; this process offers a balance of high quality, high efficiency, controllability, and scalability. Although many breakthroughs have been achieved using the molten salt-assisted metal oxide precursor (Zhou et al., 2018a), there is a concern that the introduction of salt may result in the formation of impurities in the as-synthesized product. Group VB MTMDs grown using these precursors are yet to be truly demonstrated in terms of feasibility as electrocatalysts. Moreover, the commonly used powder vaporization routes using transition metal chlorides with low melting point (e.g., TaCl_5_, VCl_3_, and S powders) are relatively limited in terms of the continuous and constant supply of precursors during the process. These restriction affects the reproducibility and controllability of the composition and thickness because there is a possibility that various intermediate compounds (e.g., V_3_S_4_, VS_2_) self-intercalated by ordered M atoms within the van der Waals gaps of group VB MTMDs will be produced (Oka et al., 1978; Zhao et al., 2020). More investigation on the role of the various growth parameter and fundamental catalytic properties of fabricated electrocatalysts will be required to facilitate the controllable, scalable, and direct fabrication of group VB MTMDs as electrocatalysts using CVD. For example, Wu et al., discovered that the dangling-bond-free surface of 2D TMDs substrates ensure a minimized diffusion barrier for the precursor atoms in the group VB MTMDs, causing the reactant atoms to migrate to the edge of the growing 2D materials (Wu et al., 2019). They obtained ultrathin group VB MTMDs with thickness as low as 1.0 nm using the 2D substrate effect. Zhao et al. successfully controlled the synthesis of TaS_2_ compounds via self-intercalation method by adjusting the Ta/S ratio (Zhao et al., 2020). DFT calculations were performed to evaluate the thermodynamic stabilities of the intercalated phases. It was found that stoichiometric H-phase TaS_2_ was formed only under S-rich conditions, whereas at higher Ta:S flux ratios, various Ta-intercalated Ta_x_S_y_ compounds attained a thermodynamically stable state. In the far future, compared to the solution-based approach, their high processing cost will be the most important issue that would need to be addressed in terms of economic viability with regard to precursors, facilities, and substrates in future developments.

### Durability of MTMDs-based electrocatalysts

The instability of MTMDs that leads to decreased catalytic performance, operational stability, and lifetime of MTMD-based electrocatalysts remains a crucial issue. The instability is primarily attributed to a chemical reaction with H_2_O/O_2_ in the ambient environment (Voiry et al., 2013a) and mechanical peeling from the electrode during operation. In general, one is the encapsulation using h-BN (Lee et al., 2015), polymer (N’diaye et al., 2012), and oxide compounds (Wood et al., 2014) to improve stability of MTMDs in electronics and optoelectronics applications; however, the introduced encapsulation layer increases the resistance of the catalysts and suppress catalytic active sites. A suitable strategy for the field of electrocatalysts should be developed to use MTMDs as electrocatalysts without structural degradation. Recently, stable self-supported electrocatalysts that can directly serve as working electrodes have attracted considerable research attention; this strategy can not only avoid ingredients from detaching from the electrode to the release H_2_ molecules (Yu et al., 2021) but also enable fast charge transfer induced by the strong interlayer interaction between the substrate and the catalysts (Kim et al., 2016). Thus, new synthetic strategies are required to directly fabricate the MTMDs-base electrocatalysts on highly conductive substrates without reacting to the electrolyte.

### Optimization for the catalytic performance of MTMDs

State-of-the-art HER electrocatalysts fabricated by MTMDs in laboratory-scale systems are summarized in Tables 3 and 4. There are clear pathways forward to enable efficient electrocatalysts, as demonstrated by group VIB STMDs-based electrocatalysts (Li et al., 2016b; Ye et al., 2016). Likewise, the rational design for the best performance of MTMDs materials could be a combination of edge, defect, and interfacial engineering. Recent progress in engineered pure MTMDs shows that these electrocatalysts exhibit superior catalytic performance and stability compared with those of STMDs and other non-precious metal electrocatalysts (such as transition metal-phosphides (Popczun et al., 2013), carbides (Dong et al., 2018), and nitrides (Xiong et al., 2017)). However, they still suffer from lower performance in comparison to Pt-based groups. One of the key strategies for the future development of MTMDs is the design of the crystal structure because there is a close correlation between the electronic structure, stability, and efficiency. In this regard, the search for new ternary MTMDs (Rezaie et al., 2021), alloying with metals (Hu et al., 2019), doped-MTMDs (Li et al., 2017b; Huang et al., 2019b), and composites based on MTMD and other TM-based materials (Wei et al., 2022) should be attempted. In particular, this method can be a major solution for MTMDs with a relatively lower performance in alkaline solutions. For instance, MTMDs with the incorporation of transition metals, such as Ni-Co-based metallic MoS_2_ (Li et al., 2017b) and NiO-1T MoS_2_ (Huang et al., 2019b) or MoS_2_/MXene/CNT ternary composites (Wei et al., 2022), exhibit superior HER activity. Furthermore, MTMDs are expected to be useful as co-catalysts for solar-driven water splitting. Although there are still few reports on the same, the high electrical conductivity and many active sites of MTMDs can indirectly affect the performance of the light absorber by reducing the recombination rate of electron-hole pairs, as well as the HER activity (Zhang et al., 2021). Recently, heterostructures such as 1T-MoS_2_/g-CN (Xu et al., 2019), 1T-MoS_2_/p-Si (Ding et al., 2014), VS_2_/g-C_3_N_4_ (Shao et al., 2018), and NbS_2_/p-Si (Gnanasekar et al., 2019) with an appropriate interface to reduce the charge transfer resistance have been reported (Table 5).

**Table 5:** MTMDs for solar-driven hydrogen production

	Synthesis	Photocatalysts	Co-catalyst	Substrate (direct growth or transfer)	Electrolyte	H_2_ rate (μmol g^−1^ h^−1^)	Photo-current (mA cm^−2^ at 0V vs RHE)	Apparent quantum efficiency (AQE)	stability	Ref.
Photocatalysts	Li-intercalation	g-CN	1T-MoS_2_	N/A	10 vol % Triethanolamine (TEOA)	5620	N/A	8.2% (420 nm)	4 cycles for 8 h	(Xu et al., 2019)
	Hydrothermal	g-C_3_N_4_	VS_2_	N/A	10 vol % Triethanolamine (TEOA)	87.4	N/A	5.5% (420 nm)	7 cycles for 14 h	(Shao et al., 2018)
PEC	CVD	p-type Si	1T-MoS_2_	p-type Si (transfer)	0.5 M H_2_SO_4_	N/A	17.6	N/A	N/A	(Ding et al., 2014)
	CVD	Si nanowire	NbS_2_	p-type Si (direct growth)	0.5 M HClO_4_	N/A	26	N/A	For 10,000s	(Gnanasekar et al., 2019)
